# Discovering Genetic Interactions in Large-Scale Association Studies by Stage-wise Likelihood Ratio Tests

**DOI:** 10.1371/journal.pgen.1005502

**Published:** 2015-09-24

**Authors:** Mattias Frånberg, Karl Gertow, Anders Hamsten, PROCARDIS consortium, Jens Lagergren, Bengt Sennblad

**Affiliations:** 1 Atherosclerosis Research Unit, Department of Medicine, Solna, Karolinska Institutet, Stockholm, Sweden; 2 Department of Numerical Analysis and Computer Science, Stockholm University, Stockholm, Sweden; 3 Science for Life Laboratory, Stockholm, Sweden; 4 School of Computer Science and Communications, KTH Royal Institute of Technology, Science for Life Laboratory, Swedish e-Science Research Centre, Stockholm, Sweden; Stanford University School of Medicine, UNITED STATES

## Abstract

Despite the success of genome-wide association studies in medical genetics, the underlying genetics of many complex diseases remains enigmatic. One plausible reason for this could be the failure to account for the presence of genetic interactions in current analyses. Exhaustive investigations of interactions are typically infeasible because the vast number of possible interactions impose hard statistical and computational challenges. There is, therefore, a need for computationally efficient methods that build on models appropriately capturing interaction. We introduce a new methodology where we augment the interaction hypothesis with a set of simpler hypotheses that are tested, in order of their complexity, against a saturated alternative hypothesis representing interaction. This sequential testing provides an efficient way to reduce the number of non-interacting variant pairs before the final interaction test. We devise two different methods, one that relies on *a priori* estimated numbers of marginally associated variants to correct for multiple tests, and a second that does this adaptively. We show that our methodology in general has an improved statistical power in comparison to seven other methods, and, using the idea of closed testing, that it controls the family-wise error rate. We apply our methodology to genetic data from the PROCARDIS coronary artery disease case/control cohort and discover three distinct interactions. While analyses on simulated data suggest that the statistical power may suffice for an exhaustive search of all variant pairs in ideal cases, we explore strategies for *a priori* selecting subsets of variant pairs to test. Our new methodology facilitates identification of new disease-relevant interactions from existing and future genome-wide association data, which may involve genes with previously unknown association to the disease. Moreover, it enables construction of interaction networks that provide a systems biology view of complex diseases, serving as a basis for more comprehensive understanding of disease pathophysiology and its clinical consequences.

## Author Summary

Many of our common diseases are driven by complex interactions between multiple genetic factors. Disease-specific, genome-wide association studies have been the prominent tool for cataloging such factors, by studying the genetic variation of a gene in a population and its association with the disease. However, these studies often fail to capture interactions between genes despite their importance. Interactions are notoriously difficult to investigate, because testing the large number of possible interactions using contemporary statistical methods requires very large sample sizes and computational resources. We have taken a step forward by developing a new statistical methodology that significantly reduces these requirements, making the study of interactions more feasible. We show that our methodology makes it possible to study interactions on a large scale without compromising the statistical accuracy. We further demonstrate the utility of our methodology on data relating to coronary artery disease and discover three distinct interactions that might provides new clues to the pathophysiology. The study of genetic interactions have the potential to link disease genes together into disease networks that provide a more detailed description of the interaction between genes and how it drives the disease.

## Introduction

Cardiovascular disease, cancers, diabetes and chronic obstructive pulmonary disease, accounting for almost 60% of the causes of death 2013, globally [1], are all examples of complex diseases. A complex disease is characterized by an intricate system of interactions between genetic, epigenetic, other intrinsic factors, and environmental factors, that constitutes its pathophysiology. The genetic architecture of many common complex diseases is poorly understood. For example, the 46 robustly associated variants that have been found for coronary artery disease (CAD) only explain 10.6% of the estimated heritability; this was shown in a recent meta-analysis of almost 200,000 individuals [2]. The same pattern of unexplained, or missing, heritability is found in most common complex diseases [3]. Assuming that the estimated heritability is correct, the possible explanations for the high ratio of missing heritability include 1) a large number of causal genetic variants, each with a small effect, 2) sequence variation that is commonly excluded from analysis, e.g. copy number variation or rare variants, 3) other commonly unmeasured heritable components, e.g. heritable epigenetic modifications, and 4) interaction effects between common variants. Moreover, any combination of these explanations is plausible. In this paper we focus on the inference of interaction in genetic association studies; this is sometimes called epistasis, epistatic interaction or genetic interaction; here we will refer to it as genetic interaction or simply interaction.

Genetic interactions are characterized by two or more variants producing an unexpected phenotype that is not easily explained by the marginal effects of the individual variants. Extensive studies in model organisms have shown that genetic interactions are common phenomena [4]. The field was pioneered by Bateson [5], who studied genetic interactions in plants and chicken. More recently, synthetic lethal interactions (in which the simultaneous occurrence of two mutations, by themselves without effect, lead to cell death) have been studied extensively in yeast and *Caenorhabditis* [6–8] and interactions between quantitative trait loci have been studied in mouse, *Drosophila* and *Caenorhabditis* [9–11]. Since interactions are widespread in other organisms, it seems unlikely that such effects would not exist in humans. Furthermore, genes are linked in metabolic, regulatory and signaling pathways and it is likely that this will be reflected as interactions between variants, as has been shown for transcriptional regulation in *Drosophila* [12]. Therefore, studies of genetic interactions have a strong potential to provide important insights about disease biology—specifically, interactions reflect dependencies in pathophysiology and may allow predictions of effects (and side effects) that are relevant for disease prognosis and treatment.

Several approaches have been developed to study genetic interactions (see [13–15] for three excellent methodology reviews). In medical genetics, the prevalent tool for modeling single variant association in unrelated individuals has been generalized linear models (GLM). The advantages of GLMs are flexibility in modeling the phenotype, easy interpretation and straightforward adjustment for confounders. Although the GLM framework can model both discrete and continuous outcomes; we will, in this work, concentrate on the case-control outcome. Studies of interactions are, however, not without issues. Firstly, the identification of interactions depends on the *scale* relating the genotypes to phenotypes. Secondly, because the GLMs are fitted by iterative procedures, the computational burden is high. Thirdly, straightforward multiple testing correction leads to low statistical power. We now elaborate briefly on these three issues.

The dependency of GLMs on a *scale* sometimes causes confusion [16, 17]. The scale is determined by a link function that maps the phenotype to the linear predictors. For example, for two predictors *a* and *b*, the phenotype *y* can be determined by an additive (*y* = *a* + *b*) or by a multiplicative (*y* = *e* ^*a*+*b*^) model. A commonly used link function in case/control studies is the logit, which is used in logistic regression. This displays a combination of mathematically favorable properties: it models the case/control selection bias, the parameters have minimal sufficient statistics, and it is the maximum entropy null model [18]. However, the choice of scale is to a large extent a modeling issue and should not be based on mathematical convenience alone. For example, when, for a set of variants, the presence of a risk allele in any single variant is sufficient to cause the disease, the log-complement link function yields an appropriate model [17, 19]. Ultimately, the best choice of scale depends on the unknown biological model that has generated the data. The choice of scale is very problematic because, even if the true model underlying the data displays interaction, it is often possible to select a scale that diminishes the interaction effect [20]. Conversely, if the true model does not display interaction, then there is another scale that, in the asymptotic case, will display interaction [17]. In response to this, Knol and VanderWeele [21] suggest that the p-value of an interaction should be reported on a set of reasonable scales to show whether the interaction seems invariant of scale. We follow this suggestion and, furthermore, extend it by constructing a test for interaction that is invariant over a set of link functions.

A different approach builds on the rationale that if, for interacting variants, certain combinations of alleles affect disease risk, then this would be reflected in differential enrichment for these allele combinations between cases and controls, and therefore a difference in their linkage coefficients (*LD*_*cases*_ and *LD*_*controls*_). The *LD*-contrast test [22] compares the normalized difference of *LD*_*cases*_ and *LD*_*controls*_ as a *χ* ^2^-distributed statistic for interaction. A recently been proposed version of the *LD*-contrast test [23] uses genotypes recoded to a pair of binary variables (according to model of inheritance).

A third approach, the multi-factor dimensionality reduction (MDR) [24], uses dimensionality reduction techniques to recode the 3 × 3 penetrance matrix into a binary variable that optimally classifies cases and controls. This is then evaluated by cross-validation and a permutation procedure is used to estimate significance. Several variants of MDR have been developed [25, 26].

One common approach to improve the computational complexity, has been to introduce the naive assumption that it is impossible for two variants to be simultaneously associated with the phenotype unless they interact. Examples include the method of [27], using a log-linear GLM, and many of the variations of the MDR method, including the original one [24, 25]. Under this naive assumption it is, in the GLM setting, sufficient to compare three models: two single variant association models and the saturated model, which will represent interaction. The parameters of these models can be efficiently estimated since they all have closed form solutions. Unfortunately, this simplification allows interactions to be incorrectly inferred between two variants that both are associated with a main effect, but there is no interaction (we will refer to this as *double main* association). As a consequence, genuine genetic interactions may be obscured by these *double main* associations [28]. In this work, we will focus on inference of genuine interactions.

Finally, the reduction in statistical power implied by correction for multiple tests constitutes a major limitation for performing interaction studies on a larger scale. For an investigation of interaction between all pairs of a set of *n* = 500,000 variants, a Bonferroni correction for *n*(*n* − 1)/2 tests gives a significance threshold of 4.0 ⋅ 10^−13^, which is considerably lower than the corresponding significance level 1.0 ⋅ 10^−7^ for a standard single variant analysis. The burden of multiple tests grows exponentially with the number variants involved in the tested interactions, and, henceforth, we will limit ourselves to the case of pair-wise interactions. Various screening strategies have been applied in attempts to improve power. These may use prior information that identify a smaller set of candidate variant pairs (we will investigate two such approaches in our analysis of biological data) or they may be based on the data at hand. An example of the latter include the screening test of Marchini [29], which removes variant pairs lacking a marginal effect for one or both of the participating variants. Millstein et al. [30], using a reasoning similar to the *LD*-contrast test above, suggested a *LD*_*cases*_ screening test for significant linkage enrichment among cases. However, observing that this induced a bias in the subsequent main test, they also proposed a *LD*_*cohort*_ screening test. The latter test relies on the linkage enrichment in cases also showing as a linkage enrichment of the pooled cases and controls, but formally does not use any prior information about disease state. Various combinations of screening and main tests have been proposed: marginal screen with logistic GLM main test [29], *LD*_*cohort*_ screen with logistic GLM main test [30, 31], and *LD*_*cases*_ screen with *LD*-contrast main test [23].

In this work we introduce a stage-wise multiple testing methodology that exhaustively tests all variant pairs. In this methodology, a sequence of hypotheses is considered in order of increasing complexity. Only variant pairs that cannot be explained better by a simpler hypothesis compared to the most complex hypothesis (representing interaction) are tested at subsequent stages. This is conceptually different from the screening approach by Marchini [29], which instead requires that a variant pair fits an intermediate screening hypothesis (of single marginal association) better than the simplest hypothesis (of no association) for it to be tested at the subsequent stage. Because the hypotheses considered are closed under intersection, we show, in two situations, that the family-wise error rate is controlled. Furthermore, since the models under the simpler hypotheses can be estimated efficiently, our methodology allows the use of full GLMs. The multiple testing correction is alleviated and results in a substantial increase of power compared to the Bonferroni correction. We also construct a scale-invariant test for interaction using several link functions. Furthermore, we assess a set of statistical methods for inferring genetic interactions on synthetic data and show that our methodology improves on these. Lastly, we discover three distinct interactions that are associated with CAD, of which one includes a novel locus.

## Results

### Theory

In this section, we describe our multiple testing methodology, which is aimed at large-scale pairwise interaction testing. We show that it gains additional power by separating a complex hypothesis into stages of simpler null models. We have derived two methods that rely on different assumptions, having different effects on the bounds of the family-wise error rate (FWER). We start by briefly reviewing general linear models (GLM), which we use to express our model of interaction, as well as the simple null models. Frequently, when GLMs are applied in pairwise interaction testing, FWER is bounded using Bonferroni.

#### Interaction in the generalized linear model

In the framework of generalized linear models (GLM), the penetrance *p*_*ab*_ of two given genotypes *X*_1_ = *a* and *X*_2_ = *b*, where *a*, *b* ∈ {0, 1, 2}, is modeled by

$$g\left(p_{ab}\right)=\alpha +\beta_{a}+\gamma_{b}+\delta_{ab}$$

where *β*_*a*_ and *γ*_*b*_ represents the *main effect* on the phenotype of *a* and *b*, respectively, *δ*_*ab*_ represents the *interaction effect* of *a* and *b*, and *g* is the *link function*. The link function is invertible and determines the scale. Sometimes, this model is restricted by adding constraints forcing an additive allele effect, that is, *β*_2_ = 2*β*_1_, *γ*_2_ = 2*γ*_1_, *δ*_21_ = *δ*_12_ = 2*δ*_11_, and *δ*_22_ = 4*δ*_11_. However, in the present methods we will *not* assume an additive allele effect. Of note, to avoid over-parameterization, we set *β*_0_ = *γ*_0_ = *δ*_00_ = *δ*_01_ = *δ*_02_ = *δ*_10_ = *δ*_20_ = 0.

A standard likelihood ratio test of interaction can be obtained by comparing the likelihood of this full model to that of a null model in which *δ*_*ab*_ = 0 for all *a*, *b*. In our stage-wise methodology, we will use such tests for various null models, corresponding to subsets of the parameters. The degrees of freedom are determined by the difference in the number of free parameters; for example, if we want to test whether *β*_*a*_, *γ*_*b*_ or *δ*_*ab*_ is different from 0 (i.e., the reduced model is *g*(*p*_*ab*_) = *α*), we would compare against a *χ* ^2^ distribution with 9 − 1 = 8 degrees of freedom.

#### Stage-wise testing for interactions

We now describe how to apply our GLM to a stage-wise test of interaction to a set *S* of multiple variant pairs. We test the interaction hypothesis by a stage-wise comparison of the full model

- *H*_*A*_ : *g*(*p*_*ab*_) = *α* + *β*_*a*_ + *γ*_*b*_ + *δ*_*ab*_

to the following null models

- *H*_1_ : *g*(*p*_*ab*_) = *α*
- *H*_2_ : *g*(*p*_*ab*_) = *α* + *β*_*a*_
- *H*_3_ : *g*(*p*_*ab*_) = *α* + *γ*_*b*_
- *H*_4_ : *g*(*p*_*ab*_) = *α* + *β*_*a*_ + *γ*_*b*_.

The *H*_1_ null model represents no association, *H*_2_ and *H*_3_ represent the cases where one single variant is associated (we will refer to this as *single main* association) and *H*_4_ represent the case where both variants simultaneously have a main effect association without interaction (*double main* association). The tests are performed sequentially. In the first stage, *H*_1_ is tested against *H*_*A*_ for all variant pairs, but subsequent stages only consider variant pairs for which the null models in previous stages have been rejected. This allows us to vary the multiple testing correction across stages, and thereby improve power compared to Bonferroni correction, as shown, below, in Results section Statistical power to detect interactions. The exact details of this correction constitute the difference between our two methods.

**Fig 1:**
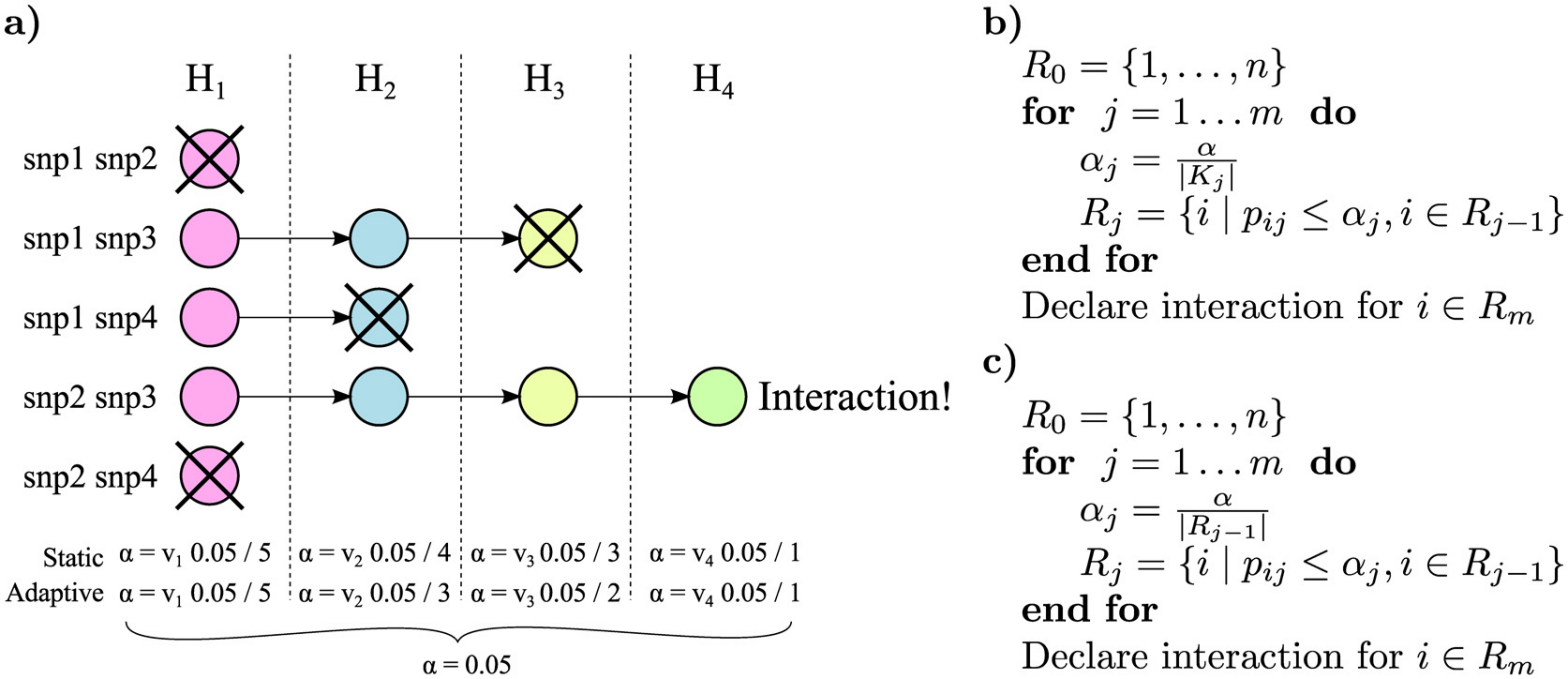
Illustration of the rejection procedure. a) We have a set of four hypotheses that are closed under intersection. We start at stage 1 by testing the simplest null hypothesis *H*_1_ for each variant pair; the p-value threshold *α* for this test is corrected for the total number of pairs. In the figure *H*_1_ is accepted in the first and last pair, and these pairs will not be tested in the subsequent stages. We then continue through the null hypotheses from simple to complex but correcting the *α* for each stage only for the expected number of pairs, in the *static* method, or the actual number of pairs, in the *adaptive* method, that are tested at this stage. Finally if all null hypotheses could be rejected for a specific pair *i* (e.g., snp2 and snp3 in the figure), we declare pair *i* to be interacting. b) Pseudocode describing the *static* stage-wise testing method. Variant pairs are ordered from 1 to *n*. Null hypotheses are ordered from 1 to *m* in any order that respects the partial order of how they are nested. Only pairs for which the null hypothesis was rejected in the previous step are considered in the current step. The p-value for testing null hypothesis *j* for pair *i* is *p*_*ij*_. Rejected hypotheses in stage *j* are contained in the set *R*_*j*_, and *α* is the significance threshold. The hypotheses of interaction for pair *i* is accepted only if the null hypotheses for all *m* tests could be rejected. c) Pseudocode describing the *adaptive* method. The overall algorithm is the same as the *static*. However, the significance threshold is now determined by the total number of rejections in the previous stage ∣*R*_*j*−1_∣.

In the first method, the *static* method, we assume that the exact number of variant pairs *belonging* to each stage is known. Intuitively, a variant pair belongs to a stage if the null model at this stage is the simplest model that is correct for the pair. To preserve the FWER, we introduce weights {*w*_*s*_, *s* ∈ [4] : ∑_*s*∈[4]_ *w*_*s*_ = 1}, one for each stage, that adjust the p-value thresholds for the four stages of tests. Let *K*_*s*_ be the number of variant pairs belonging to a stage *t* ≥ *s*, and *p*_*is*_ be the p-value of stage *s* for pair *i*. If *p*_*is*_ < *w*_*s*_ *α*/*K*_*s*_, the pair *i* is tested for stage *s* + 1. The idea is illustrated in Fig 1a and the algorithm is outlined in Fig 1b. A generalized version of the closed testing principle [32] can be used to show that this method controls the FWER, a proof is provided in S1 Text. The adjusted p-value is defined [33] and can be computed by

$${\tilde{p}}_{i}=\max_{s}\frac{K_{s}p_{is}}{w_{s}}.$$

The following is an example on how to estimate the number of hypotheses in each stage. Let *N* be the number of genotyped variants and *M* be the number of marginally associated variants (which, e.g., can be taken from a meta analysis). Then estimates of the *static* multiple testing correction, *K*_*s*_, for each stage are, in order, $\frac{N(N-1)}{2}$, *N* ⋅ *M*, *N* ⋅ *M* and $\frac{M(M-1)}{2}$.

The second method, the *adaptive* method, only corrects for the number of rejections in the previous stage. This more flexible and powerful method is tailored for situations where an estimate of the number of marginally associated variants is not available. Because the multiple testing corrections become dynamic, the closed testing theorem cannot be directly applied. We therefore need to make the additional assumption that alternative hypotheses are asymptotically rejected, which is true for any consistent test. A proof is provided in S1 Text.

The *adaptive* method is identical to the *static* method, with the exception that *K*_*s*_ is replaced by ∣*R*_*s*−1_∣ where *R*_*s*_ is the set of rejected hypotheses in stage *s*, and *R*_0_ is the total number of pairs. The algorithm is outlined in Fig 1c. A conservative estimate of the adjusted p-value is

$${\tilde{p}}_{i}=\max_{s}\frac{|R_{s-1}|p_{is}}{w_{s}},$$

since as you alter the significance threshold for a stage, different number of hypotheses, ∣*R*_⋅_∣, will be present on the following stages.

An advantage with our methodology is that *H*_1_ to *H*_3_ can be efficiently computed [27]. The tests for *H*_1_ to *H*_3_ is also invariant of the scale as shown in S1 Text. Moreover, key for the improved power of our methodology is two assumptions relating to the test for *H*_1_: (1) that it has high power to identify interactions, and (2) that it, at the same time, is effective in removing false positives and thereby reducing the severity of multiple testing in the following stages. In our simulation results assessing assumption (1), the *H*_1_ test is more powerful than that for *H*_4_, despite the increase in degrees of freedom (cf. Results section Statistical power to detect interactions). Moreover, for complex diseases, the number of associated variants identified in large meta analyses is commonly less than 100 [3], suggesting that assumption (2) will not be a bottleneck. We also investigate the practical performance of the methods.

#### Scale-invariance test

The choice of link function, *g*, has a substantial influence on any study of interactions based on GLMs. Ideally, we want the inferred interaction to be invariant to the scale, that is, the rejection the null model (of no interaction) should hold regardless of the link function used [21]. We will now describe a scale-invariant test that formalizes this notion.

With slight abuse of notation, we replace hypothesis *H*_4_ in the previous section with

- $H_{4}^{\prime }:\underset{g\in G}{⋃}\left\{g(p_{ij})=\alpha +\beta_{i}+\gamma_{j}\right\}$,

where *G* is some set of pre-selected link functions (note that the models *H*_1_, *H*_2_ and *H*_3_ are saturated and changing the link function will not have an effect). The test for $H_{4}^{\prime }$ is constructed according to the intersection-union principle [34], that is, if *q*_*s*_ is the p-value for scale *s* then the combined p-value *q* is

$$q=\max_{s}q_{s},$$

that is, the test requires significance for all link functions; we will refer to this test as the *scale invariance test*. Notice that the choice of the set *G* of link functions will depend on the study. For example, *G* may contain a single link function if there is strong prior evidence for this link function. Furthermore, the study design will influence the choices of *G*; for example, an odds ratio-based link function is unaffected by the sampling-bias introduced in case-control studies and, omission of a such a link function may lead to the wrong conclusion. Here, we investigate the impact of a set of commonly used link functions, shown in Table 1.

**Table 1:** Link functions, for a GLM with a binary outcome, used in this study.

Link	*g*(*p*_*ij*_)
Additive penetrance (identity)	*p*_*ij*_
Multiplicative penetrance (log)	log(*p*_*ij*_)
Genetic heterogeneity (log-complement)	log(1 − *p*_*ij*_)
Additive odds (odds)	$\frac{p_{ij}}{1-p_{ij}}$
Multiplicative odds (logit)	$\text{log}\;\left(\frac{p_{ij}}{1-p_{ij}}\right)$

### Statistical power to detect interactions

In this section, we will give an account of three investigations of statistical power that all indicate the utility of our stage-wise methodology. The generation of simulated data used in these investigations are described in Material and methods section Generation of synthetic data for estimation of statistical power.

**Fig 2:**
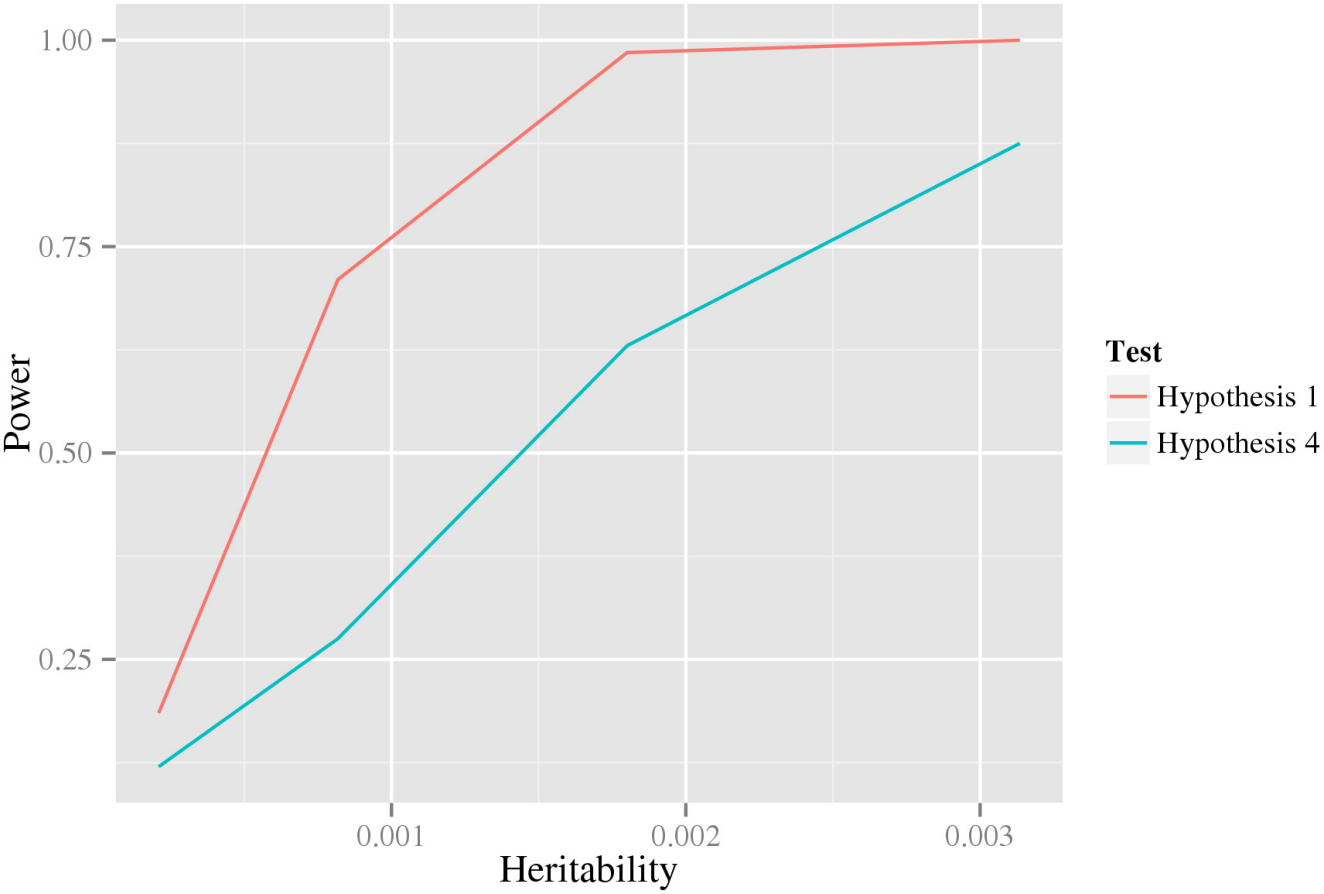
The power of the first and last test under a double dominant interaction model. The x-axis is the heritability of the model. The y-axis is the statistical power. The colored lines correspond to two different tests: the one performed in the first stage that tests the null hypothesis of no interaction, *H*_1_, (red), and the one performed in the last stage that specifically tests the interaction parameters, *H*_2_, (blue). The logit link function and a nominal significance level of 0.05 was used for the analysis.

The intuitive idea behind the stage-wise methodology is that we aim to (1) reduce the number of tests in later stages compared to earlier, while (2) asserting that actual interactions advance to later stages. We show in the Results section Analysis of biological data, below, that the number of tests in the last stage is in fact substantially reduced, suggesting that aim (1) is unlikely to be a problem. Here, we have investigated aim (2 by comparing the power of the tests in the first and last stage. That is, for data generated from *H*_*A*_, we compare the power of the likelihood ratio test of *H*_1_ against *H*_*A*_ to that of the test of *H*_4_ against *H*_*A*_. Indeed, the results in Fig 2 (using data generated from a double dominant interaction model) suggests that the test in the first stage, at least under these conditions, have substantially greater power than that in the last stage. However, the test in the first stage can obviously not be used as a test for interaction by itself, since it measures any kind of association to the phenotype, including, for example, pairs for which only one of the variants is associated.

**Fig 3:**
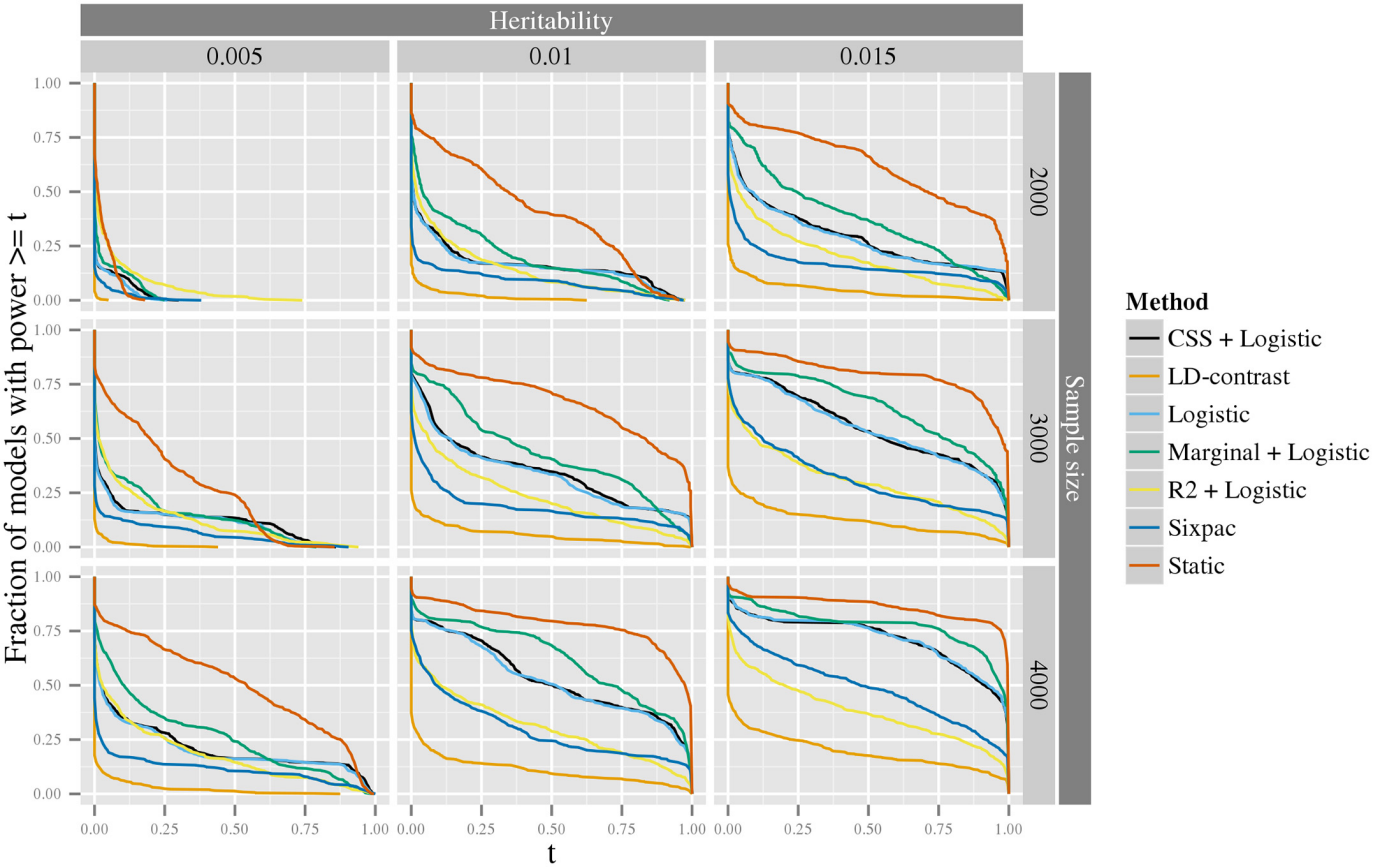
The exceedence distribution of power over all possible interaction models with a specific heritability. For each plot, the *x*-axis shows a threshold, *t*, for power to detect an interaction among 10^12^ variant pairs, and the corresponding y-axis shows the fraction of models that have a power greater than or equal to *t*. The rows correspond to the sample size of a balanced design e.g. 2000 indicates 2000 cases and 2000 controls. The columns correspond to the heritability of the models. Six methods for inference of interactions *Logistic*, *Marginal+logistic*, *CSS+marginal*, **R*^2^+marginal*, **LD*-contrast*, and *Sixpac*; see text for details), are compared to our *static* stage-wise scale-invariant method. The line colors used to denote the different methods are shown in the legend to the right.

We further investigated the distribution of statistical power of seven methods using simulated data generated from the spectrum of all possible interaction models, following the ideas of [35] (see Material and methods section Generation of synthetic data for estimation of statistical power for details). The first of these methods is our *static* method, and the remaining methods include four methods based on a logit-link GLM with different screening strategies, *Logistic* (without screening), *Marginal+logistic* [29], *CSS+logistic* [30] and **R*^2^+logistic* [31]) and two methods based on the *LD*-contrast test with different screening strategies, **LD*-contrast* (without screening), and *Sixpac* [23] (a *LD*_*cases*_+*LD*-contrast method), for details, see Material and methods section Comparison of statistical methods. It should be noted that none of the latter six methods are scale-invariant—one may expect that this property would enhance their power. For simplicity of simulations, we only evaluated the *static* method here; however, since the *adaptive* method is more powerful than the *static*, this can also be viewed as a conservative estimate of the power of the *adaptive* method. As can be seen in Fig 3, the *static* method consistently has greater power than the other approaches. The *marginal+logistic* method performs best of the remaining methods, while the the **LD*-contrast* method have the worst performance. In S1–S4 Figs, we also report the result of a more computationally intensive power comparison, including the above methods, as well as our *adaptive* stage-wise scale-invariance method and the Model-based MDR (*MB-MDR*) method [26] (see S2 Text for details). These results corroborate those above, that is, for most models our stage-wise methods performs better than the other methods (see further discussion in S2 Text).

**Fig 4:**
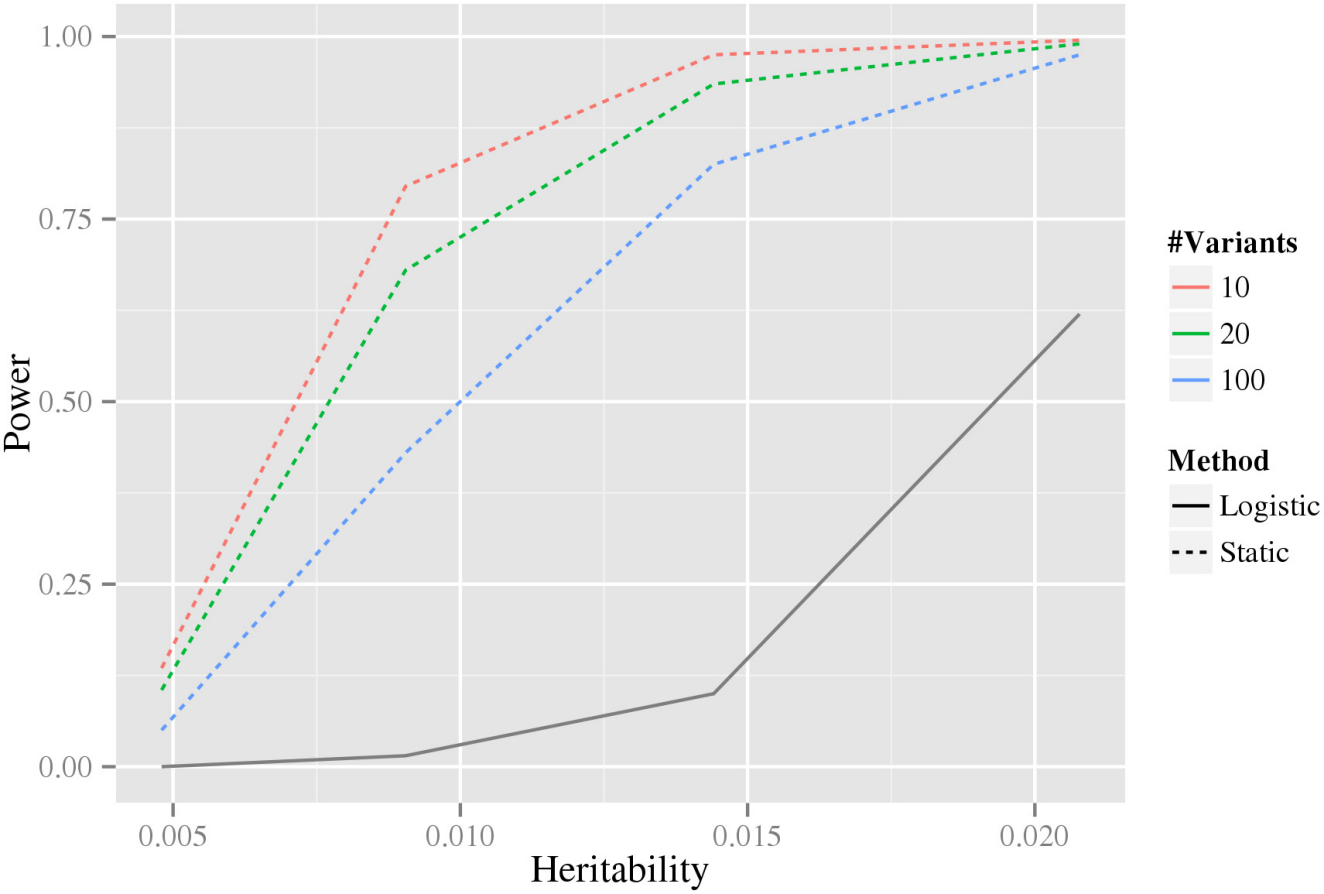
The dependence of power on the estimated number of associated variants. The x-axis is the heritability and the y-axis is the estimated power. The colored dashed lines correspond to our stage-wise test using different number of associated variants, as indicated by the legend to the right. The black solid line corresponds to the logistic regression method using Bonferroni correction (which does not depend on the estimated number of associated variants). The power estimates are based on data simulated from the double dominant interaction model.

Intuitively, when more variants are associated with the phenotype in our stage-wise methodology, the multiple testing correction in the intermediate stages becomes larger, and therefore statistical power is reduced. For this reason, we investigated how the statistical power depends on the number of associated variants using data simulated from the double-dominant interaction model (see Material and methods section Generation of synthetic data for estimation of statistical power). As shown in Fig 4, the power decreases as the number of associated variants increases. Because of the additional penalty of the weight, the *static* method can have lower power than directly testing interaction using a Bonferroni correction, precisely when *M*(*M* − 1) > *w*_4_ *N*(*N* − 1) (where *N* is the total number of variants and *M* is the number of associated variants). It can be noted that for our biological data, *M*(*M* − 1) = 306 ≪ *w*_4_ *N*(*N* − 1) = 346,035,421.8 (based on the *N* = 33,963 tested variants and the *M* = 18 robustly associated CAD variants present on the IBC-chip, cf. S1 Table).

### Family-wise error rate on the class of null models

Both the *static* and *adaptive* stage-wise methods are based on the likelihood ratio test, which is asymptotically correct. As we show in S1 Text, the *adaptive* method controls the FWER asymptotically. For the *static* method on the other hand, we can even show that the FWER is controlled for any data size. Consequently, it is interesting to investigate the behavior of both methods on finite data and compare it with that of the same seven methods as in the power comparison (*Logistic*, *Marginal+logistic*, *CSS+logistic*, **R*^2^+logistic*, **LD*-contrast*, *Sixpac*, and *MB-MDR*). We considered two cases, one close to the assumptions of our methods, and one designed to be challenging. We investigated these two cases using simulated data (see further Material and methods section Generation of synthetic data for estimation of statistical power).

In the first case, we used each of our null models, *H*_1_: no association, *H*_2_/*H*_3_: *single main* association, and five models for *double main* association (*H*_4_) with the identity, log, log-complement, odds and a logit link functions, respectively, to generate the phenotype based on a single pair of variants. The first seven rows in Table 2 show that both the *static* and *adaptive* methods accurately control the FWER under these circumstances (i.e., FWER is below or close to the expected value of 0.05). All seven other methods control the FWER for the no association and *single main* models. However, for the *double main* models, they control FWER only on the multiplicative scale (i.e., with the log, logit and log complement link functions). For the remaining models (*double main*:identity and *double main*:odds) these methods fail to control FWER, with the exception of the **R*^2^+logistic* that controls FWER for the *double main*:identity model.

**Table 2:** Estimated family-wise error rate for all methods under different null models, using a significance threshold of 0.05.

**Model** ^1^	**Adaptive**	**Static**	**Logistic**	**Marginal+logistic**	**CSS+logistic**	***R*^2^+logistic**	***LD*-contrast**	**Sixpac**	**MB-MDR**
No association	0.005	0.005	0.005	0.015	0.02	0.015	0.045	0.005	0.015
Single^2^	0	0	0.02	0.015	0.025	0.05	0.045	0.005	0.02
Double^3^:identity	0.02	0.025	0.275	0.355	0.145	0.075	0.08	0.22	0.31
Double:log	0.01	0.01	0.025	0.035	0.035	0.035	0.03	0.01	0.035
Double:odds	0.035	0.02	0.955	0.975	0.54	0.16	0.66	0.985	0.99
Double:logit	0.015	0.015	0.04	0.03	0.035	0.07	0.035	0.005	0.035
Double:log-compl	0.03	0.025	0.03	0.045	0.035	0.035	0.03	0.01	0.04
Multi^4^:10	0.15	0.015	0.035	0.03	0.025	0.05	0.055	0.015	0.06
Multi:20	0.145	0.015	0.025	0.05	0.025	0.07	0.055	0.005	0.055
Multi:30	0.135	0.035	0.01	0.03	0.03	0.025	0.045	0	0.015

^1^ Model from which data was generated

^2^ Single main association

^3^ Double main association

^4^ Multivariate additive GLM

In the second case, where we attempted to construct instances that challenge the additional asymptotic assumptions made in the *adaptive* method. The phenotype was here determined by an multivariate additive GLM with logit link function on a set of *L* ∈ {10, 20, 30} markers. The parameter distributions were chosen with the intention to let only a small and difficult subset of the variant pairs to reach the stage they belong to. The last three rows in Table 2 show that the *static* method controls the FWER, but suggest that for the *adaptive* method, FWER is inflated by approximately a factor 3 compared to the desired rate. The remaining methods controls FWER in this setting, possibly an effect of the data being generated on a multiplicative scale.

### Analysis of biological data

We applied our stage-wise methodology on genome-wide CAD case-control data from the PROCARDIS study, using our five link functions. To enhance such a large-scale analysis, we explore three strategies for selecting subsets of variant pairs to test. The first strategy represents a genome-wide approach, while the latter two strategies were designed to a priori enrich for pairs likely to exhibit interaction. For the same reason, our main focus will be on the more powerful adaptive method, combined with validation of any significant discoveries in a separate cohort.

In the first strategy all 229,050,992 pairs, for which the product of the minor allele frequencies > 0.04, were selected. The stage-wise methodology subsequently reduced the number of pairs to 15269, 7712 and 93. This analysis resulted in seven variant pairs that were significant for at least one link function, see Table 3. We used genomic proximity to coarsely estimate genes corresponding to these variant pairs. One variant pair, indicating an interaction between IL1R1 and CDNK2B-AS1, was significant on the additive odds scale, only. The p-values for the other scales were quite far from significance, indicating that this association is not scale-invariant. In other words, this interaction should be interpreted with care, as we cannot exclude the possibility that this is the effect of *double main* association, e.g., on the logit scale, without interaction. The remaining six variant pairs indicated an interaction between the genes MIA3 and CDNK2B-AS1. None of these passed the scale-invariance test (i.e., was significant for all link functions). However, for most of these variant pairs, the p-values for all scales are of the same magnitude and reasonably close to the significance level of 0.05 (see, e.g., the rs4846770–rs518394 variant pair), perhaps suggesting that this could be an effect of insufficient power rather than scale dependency.

**Table 3:** Significant pairs using different strategies.

**All vs all strategy**				Odds		Penetrance		
SNP 1^1^	SNP 2^1^	Locus 1^2^	Locus 2^2^	Add^3,4^	Mul^3,5^	Add^3,6^	Mul^3,7^	Het^3,8^
rs3917245	rs1412832	IL1R1	CDKN2B-AS1	**0.03746**	0.1463	0.1412	0.2855	0.07222
rs4846770	rs518394	MIA3	CDKN2B-AS1	0.06048	**0.03391**	**0.03419**	**0.0299**	**0.0468**
rs17163313	rs518394	MIA3	CDKN2B-AS1	0.08093	**0.04386**	**0.04438**	**0.03136**	0.0617
rs17163313	rs2069418	MIA3	CDKN2B-AS1	0.1422	0.06734	0.06773	**0.04921**	0.1015
rs2378584	rs518394	MIA3	CDKN2B-AS1	0.08615	**0.04893**	**0.04929**	**0.04398**	0.06707
rs4846770	rs2069418	MIA3	CDKN2B-AS1	0.09625	**0.04741**	**0.04753**	**0.04296**	0.06983
rs17163301	rs518394	MIA3	CDKN2B-AS1	0.08487	**0.04869**	**0.04899**	**0.0457**	0.06637
**CAD loci vs all strategy**				Odds		Penetrance		
SNP 1	SNP 2	Locus 1	Locus 2	Add	Mul	Add	Mul	Het
rs17465637	rs518394	MIA3	CDKN2B-AS1	0.08232	**0.04734**	**0.04769**	**0.03447**	0.06405
**HumanNet strategy**				Odds		Penetrance		
SNP 1	SNP 2	Locus 1	Locus 2	Add	Mul	Add	Mul	Het
rs4694178	rs583104	IL8,CXCL6	PSRC1	**0.04876**	**0.04876**	**0.04876**	**0.04876**	**0.04876**
rs4694178	rs602633	IL8,CXCL6	PSRC1	**0.006329**	**0.006329**	**0.006329**	**0.006329**	**0.006329**

^1^Variant pairs that are statistically significant on at least one scale.

^2^The loci most close, in genomic proximity, to the corresponding variant.

^3^Adjusted p-values; significant p-values are indicated by bold face; significance on all scales is required to pass the scale-invariance test.

^4^Additive odds ratio

^5^Multiplicative odds ratio

^6^Additive penetrance

^7^Multiplicative penetrance

^8^Genetic heterogeneity

In the second strategy 314,445 pairs were selected based on loci previously associated with CAD. This is based on the common hypothesis that some robust CAD associations may be the marginal effects of interacting variant pairs. Candidate pairs were formed by taking each of the previously associated CAD variants, see S1 Table, and combining it with each other variant. Interestingly, similar to the results from the all-vs-all strategy above, one variant pair indicating an interaction between MIA3 and CDNK2B-AS1 was significant for several link functions, but again, just, failed the scale-invariance test, see Table 3. Somewhat unexpectedly, this variant pair did not coincide with any of those in the all-vs-all analysis. However, it turns out that, while variants for both MIA3 and CDNK2B-AS1 have previously been robustly associated to CAD (see S1 Table), these variants did not include any member of the top-scoring variant pairs in the all-vs-all analysis. This enrichment strategy might therefore have been suboptimal.

In the third strategy, we used prior information from HumanNet [36], a probabilistic functional gene network that links genes for which significant evidence of interaction have been provided in one or more omics experiments; this resulted in 2,319,906 variant pairs. We found two variant pairs that were significant for all five link functions, thereby passing the scale-invariance test, see Table 3. For each of these two pairs, genomic proximity suggests that an interaction between PSRC1 and CXCL6 is associated to CAD, and, thus, may play a role in its pathophysiology. The exact mechanism of the interaction is, however, unknown, and the evidence for it in HumanNet was merely reported as co-expression between human genes.

The maximal effect size for each discovered interaction range from 0.4718 to 1.379 (S2 Table); as a reference, the effect sizes for robustly CAD-associated single variants are commonly around 0.285 [37]. While, after adjustment for age, sex, smoking, and population stratification, most effect sizes were reduced, this was not the case for the CXCL6-PSRC1-related interactions (see S3 Table). The penetrance pattern of one of the CXCL6-PSRC1 variant pairs, rs4694178 and rs602633, is shown in S5 Fig. Of note, it shows a marked directional change in risk for individuals carrying the major rs4694178 homozygote and the minor rs602633 homozygote.

We then investigated the reproducibility of the CXCL6-PSRC1-related interaction on a non-overlapping sub-cohort of PROCARDIS. This sub-cohort consists of 1797 cases and 2677 controls, which were genotyped on the Illumina Human1M Quad chip. The exact variants of the significant pair were not genotyped, and was therefore imputed (and hard-called) using the 1000 Genomes phase 3 reference panel. We tested interaction directly using a GLM combined with our link functions. This resulted in the p-values 0.174, 0.241, 0.103, 0.056, 0.156, for the identity, log, log-complement, odds and logit link functions respectively. We note that, while the p-value for the odds scale is close to significance, the replication clearly did not pass the scale-invariance test. Despite this, the penetrance patterns for different allele combinations were very consistent between discovery and replication analyses, compare S5 and S6 Figs. Of note, is that the minor allele frequency for rs602633 is relatively different between the two cohorts, see S4 Table. We, furthermore, expanded the search to the ten closest variants on both sides of both significant variants. The best variant pair, rs11730560 and rs1277930, reached nominal significance, and the p-values were 0.023, 0.0311, 0.014, 0.0084, 0.0209, again for the identity, log, log-complement, odds and logit link functions respectively. It did, however, not pass multiple testing accounting for all the 380 tested variant pairs.

We also performed analyses using the *static* method assuming 100 marginally associated variants with the same search strategies, but no variant pair was significant on any scale for any of the strategies. This may be a consequence of the expected lower power of the *static* method.

## Discussion

We have introduced a new stage-wise methodology that is statistically and computationally efficient for large-scale inference of genetic interactions. We have derived two separate methods: The first is the *static* method that uses *a priori* estimated multiple testing correction factors; here we have used the number of published robustly associated CAD SNPs to obtain such an estimate. The second *adaptive* method does not rely on the assumption of known correction factors, but uses the number of associated variant pairs at each stage to compute the multiple testing factors. To the best of our knowledge, this is the first method that uses the idea of a closed set of hypotheses to perform an exhaustive pairwise scan of interactions. We have shown that this stage-wise method performs better on a large number of interaction models compared to other statistical methods. The basic idea is that instead of directly testing all possible variant pairs for interaction, we use a sequence of more general association tests as a filter to reduce the number of pairs until only potential interactions remains. This shifts much of the multiple testing burden from the final interaction test to the preceding general tests. Because the tests leading up to the interaction test in general are more powerful (i.e., interactions will not be discarded), this results in higher overall power.

Our simulation results show that our new methods in general have higher statistical power than other common interaction inference methods. For certain specific models and low MAFs, the *Sixpac* method [23] perform relatively well, but its performance over the spectrum of all possible interaction models is low. The simulations suggest that, in ideal cases, it may be possible to infer interactions using our stage-wise methodology even when correcting for 10^12^ pairs, since each stage greatly reduces the number of tested interactions. However, we conjecture that, in practice, it will be important to take advantage of prior information in order to reduce the number of tested interactions; for example, we used information from the HumanNet database to select candidate interactions. Moreover, the methodology presented in this paper can also be combined with screening procedures such as *LD*_*cohort*_ [30, 31] or the efficient *probable approximate complete search* algorithm of [23]. This may give even further gains in power and computational speed.

Deciding which scale to work on (i.e., which link function to use, see Table 1) can be troublesome and many researchers advocate a favorite scale for statistical or biological reasons. Testing on a single scale will improve the statistical power for interactions that fit that scale compared to testing multiple scales. However, if pairs of variants are additive on another scale, this approach will lead to an increased number of false positives, in the sense that there exists simpler models that explain the data. In our framework we offer a compromise: we display all pairs that are significant on at least one scale, but also provide a test that require significance on multiple scales. In this way, a researcher can interpret the significance of an individual scale in the context of the other scales. From our analyses of biological data, no particular scale appear to consistently be the critical one for the scale-invariance test.

We note that the scale-invariance test provides an advantage in terms of FWER control. While most other methods failed to control FWER for data generated with a link function that was sufficiently dissimilar from that underlying the method, the scale-invariance test allowed our methods to control FWER for data generated with any tested link function. Although the *static* method could be derived using closed testing, the derivation of the *adaptive* method relied on additional assumptions that may be difficult to satisfy in practice. We observed that this could cause inflation of the FWER under a specifically designed additive model with multiple weakly associated variants. We note that, while analytically straight-forward to work with, the FWER is known to be a conservative control of the experimental error at the expense of power [38]. One future direction could therefore be to investigate other error control measures, for example the false discovery rate (FDR) [38]. Moreover, there are several cases where the advantages, in terms of computational efficiency and statistical power, of the *adaptive* method may compensate for a relatively modest inflation in the FWER. Specifically, as validation is conventionally required in genetics studies, the *adaptive* method can be used as a powerful tool in the discovery phase of large-scale studies.

Our biological analysis identifies the well known CDKN2B-AS1 locus, or ANRIL, which encodes an anti-sense RNA [39]. The region contains several variants that are robustly associated with CAD but the pathophysiology of ANRIL is unknown. Interestingly, we detect an interaction between CDKN2B-AS1 and MIA3, another established CAD locus [2], potentially indicating a new lead on CAD pathophysiology. Variants in the CELSR2-PSRC1-SORT1 gene cluster have previously been shown to be associated to CAD and lipid traits [2], although the exact causal relation of the genes is unclear. Our results suggest that HumanNet’s co-expression-based connection between CXCL6 and PSRC1 in fact mirrors a genetic interaction in CAD, supporting a role of PSRC1 in CAD (in line with recent results [40]). Moreover, inflammation has long been seen as an important component of the pathology of atherosclerosis, but few inflammation genes have been implicated by genome-wide association studies [41], and only in meta-analyses. It is therefore interesting that in the two sets of variant pairs unbiased with respect to CAD, we find interactions involving genes clearly implicated in regulation of inflammation, i.e., the interleukin- and chemokine-related genes IL1R1 and CXCL6 (IL8). Of course, follow-up functional investigations are required to fully understand the potential pathophysiological consequences of these interactions.

Complex diseases are multi-gene and multi-factorial diseases characterized by complex interactions between genetic, regulatory, metabolic and environmental factors. The majority of complex disease genome-wide association studies have employed traditional association analyses of single genetic markers, which only have been able to explain a small fraction of the disease heritability. A perhaps more conclusive approach would be to reconstruct the complex dependencies between factors as an interaction network reflecting the disease pathophysiology. This approach, however, has so far been hampered by the lack of efficient methods for inference of interactions associated to disease. The *static* and *adaptive* methods are two effective ways to discover genetic interactions, and the flexibility of GLMs allows them to be applied to a wide range of different phenotypes. Genetic interactions, and in particular the construction of interaction networks explaining the pathophysiology of the disease, have a potential for clinical relevance, both in terms of prognosis, treatment and drug development. The ideas of stage-wise testing is furthermore applicable outside medical genetics, whenever a large number of complex hypotheses are tested.

## Materials and Methods

### Ethics statement

The PROCARDIS study was carried out in accordance with the Helsinki Declaration and approved by the Institutional Review Board (IRB) at each one of the 4 recruiting centers: the Regional Ethics Review Board at Karolinska Institutet, Stockholm in Sweden (approval number 98-482 and 03-491), the IRB at the University of Munster, Munster, in Germany, the IRB at the Mario Negri Institute, Milano in Italy and the IRB at the University of Oxford, Oxford, United Kingdom. All study participants provided their written informed consent to participate in the study, a procedure approved by each one of the local ethical committees.

### Biological data—the PROCARDIS cohort

A subset of the PROCARDIS cohort has previously [42] been genotyped with the Illumina IBC chip, a iSelect Custom Genotyping BeadChip [43]. This chip contains 48,742 variants in approximately 2,100 candidate genes that are believed to be involved in vascular disease processes. The subset of PROCARDIS used in this study are 3,162 cases and 3,353 controls of which 3,865 are males and 2,650 are females. The disease phenotype is CAD (including myocardial infarction). Multidimensional factor analysis indicated no significant population structure.

The following quality control was performed. We removed variants with a minor allele frequency < 0.05, with significant deviation from Hardy-Weinberg equilibrium *p* < 10^−6^, and removed the variants from the X-chromosome to avoid confounding with gender, leaving us with 33,963 variants.

We performed simulations of all possible weight combinations with a precision of 0.1, the results can be seen in S5 Table. The choice seems to have little impact, and the best weight combination was 0.1, 0.3, 0.3 and 0.3, which is the one we used on biological data.

#### Strategies for the selection of variant pairs to test

We have considered three *a priori* determined strategies for selecting sets of variant pairs to investigate for genetic interaction. Firstly, we considered all possible pairs of variants. We removed pairs for which the product of the minor allele frequencies was less than 0.04, as the likelihood ratio approximations become unstable for small minor allele frequencies. We also removed pairs in which the variants were less than 1 Mbp from each other, since the GLM models fail in presence of LD. Secondly, we considered pairs that contain at least one previously robustly associated variant. These robustly associated variants were taken from 46 published CAD-associated variants found in large-scale meta analyses [2], 18 of these were available on the IBC chip and can be found in S1 Table. Thirdly, we considered variant pairs whose corresponding genes have an edge in the HumanNet interaction database [36]. We downloaded HumanNet version 1.0 from the HumanNet web page. The genes in HumanNet is indexed by UniProt Ids and we therefore associated UniProt IDs to variants, as follows: Variants were annotated with ANNOVAR [44] and mapped to their nearest genes by genomic proximity; if there were more than one then the variant was mapped to both. We downloaded Uniprot IDs for all genes that were downloaded from Ensembl Biomart [45]. We then combined this information to assign each variant to one or more Uniprot IDs. A variant pair was then formed if the corresponding genes had an edge in HumanNet.

### Generation of synthetic data for estimation of statistical power

We used two different simulation strategies for the power estimation. The first of these was used to compare the stage-wise scale-invariance method to other methods, see further next section. Models were constructed by enumerating all possible penetrance matrices displaying interaction for a single variant pair [35], as follows: The models were initially restricted to complete penetrance, that is, the penetrance is either 0 or 1, which allowed us to enumerate all 2^9^ = 512 penetrance matrices. Only models considered to interact were included, here a model was defined as an interaction if the penetrance matrix could not be decomposed according to Risch’s [19] definition of genetic heterogeneity. That is, formally, let *P* be 3 × 3 binary penetrance matrix. Then *P* is *not* an interaction if and only if there exists two 3 × 3 binary matrices, *R* with identical rows, and *C* with identical columns, and *P* cannot be written as the logical OR between *R* and *C*. The genetic heterogeneity definition was chosen because it excludes most marginal effect-only models, thereby reducing noise in the power estimation, and because it can easily be evaluated for complete penetrance matrices. The penetrance matrix was then reduced to continuous values by changing the 0’s to a specified base risk of *β*_0_ and the 1’s to *β*_0_ + *β*_1_. To enhance comparison of models, we used heritability, *H* ^2^, as a summary measure of all genetic effects in a model, where heritability was defined as

$$H^{2}=\sum_{i,j}\frac{{\left(\text{Pr}\left(Y=1\right)-\text{Pr}\left(Y=1|X_{1}=i,X_{2}=j\right)\right)}^{2}\text{Pr}\left(X_{1}=i,X_{2}=j\right)}{\text{Pr}(Y=1)(1-\text{Pr}(Y=1))}.$$

For each model, the parameter *β*_1_ was adjusted to obtain heritabilities of 0.005, 0.010 and 0.015. Using this enumeration we obtain a set of models, each defined by a matrix of penetrances for each genotype combination Pr(*Y* = 1∣*X*_1_ = *i*, *X*_2_ = *j*) (cf. S7, S8 and S9 Figs and S2 Text). The genotypes for cases and controls were then generated using Bayes’ theorem

$$\text{Pr}\left(X_{1}=i,X_{2}=j|Y=1\right)=\frac{\text{Pr}\left(Y=1|X_{1}=i,X_{2}=j\right)\text{Pr}\left(X_{1}=i\right)\text{Pr}\left(X_{2}=j\right)}{\sum_{k,l}\text{Pr}\left(Y=1|X_{1}=k,X_{2}=l\right)\text{Pr}\left(X_{1}=k\right)\text{Pr}\left(X_{2}=l\right)}$$

to get the multinomial distribution over genotypes. We generated 1,000 data sets from each of these models. We assumed a balanced design (i.e., same number of cases and controls), the sample size for each group was varied over 2,000, 3,000, and 4,000, the heritability was varied over 0.015, 0.020 and 0.025, and the minor allele frequency was fixed to 0.3. Each data set comprised a single interacting variant pair, and to model multiple testing, we assumed that there were 10^6^ variants and 10^12^ variant pairs tested. For each model and each parameter combination, the power of a method to detect interaction was estimated over the 1,000 replicates. The method’s power over the spectrum of tested interaction models were then summarized in an exceedence plot.

We performed two additional power analyses using a second simulation strategy, where we used data simulated from a specific interaction model, the double dominant model (described in S2 Text.1), in which *α* = *β*_1_ = *β*_2_ = *γ*_1_ = *γ*_2_ = 0 and *δ*_11_ = *δ*_12_ = *δ*_21_ = *δ*_22_ = *x*, and a logit link function was used. The value *x* was then varied to get the heritability 0.01, 0.02 and 0.03. This analysis used a fixed sample size of 3,000 cases and 3,000 controls. The minor allele frequency was set to 0.3 at both loci.

In the first of these two power analyses, we investigated how the relative power in detecting an associated variant pair generated under an interaction model varies over the different individual stages in the stage-wise approach, specifically we compared the power in the first and the last stages (i.e., using the null models *H*_1_ and *H*_4_). The parameters of the double dominant model can be seen in S6 Table.

In the second power analysis, where we studied the power of the *static* method to detect an interacting pair as a function of the estimated number of marginally associated variants, we set the total number of variants tested, *N* = 1,000,000, and the number of marginally associated variants was varied, *M* ∈ {10, 20, 100}. For the *static* method the corrections for each stage, in order, then was set to *N*(*N* − 1)/2, *M* ⋅ *N*, *M* ⋅ *N* and *M* ⋅ (*M* − 1)/2. The parameters of the double dominant model can be seen in S7 Table.

### Generation of synthetic data for FWER estimation

The FWER estimation was based on simulated data. We generated data from ten different null models representing two different cases: The first case corresponds to the null models in our stage-wise methodology: no association, *single main* association and five null models with *double main* effects corresponding to the link functions in Table 1; the second case represents a more challenging scenario and comprise three null models with multiple main effects.

For each null model we generated 200 data set replicates that contained 500 − *L* unassociated and *L* associated variants, where *L* depends on the null model, *L* = 0 for the no association, *L* = 1 for the single association, *L* = 2 for the double main models, and *L* = 10, 20, 30 for the multivariate model. For each data set there was therefore *L* associated variants according to the null model. For each replicate this resulted in 124,750 pairs. The minor allele frequency was sampled uniformly between 0.2 and 0.4. We sampled individuals until we obtained 4000 cases and 4000 controls. The parameters used in each null model can be seen in S8 Table.

For the null models with multiple main effects, we used an additive logistic regression model to generate the phenotype. Let *L* ∈ {10, 20, 30} be the number of variants to include from the chromosome, then the model was defined

$$\log(\frac{p}{1-p})=\beta_{0}+\sum_{i=1}^{L}\beta_{i}x_{i}$$

where *x*_*i*_ ∈ {0, 1, 2} and *β*_*i*_ ∼ *N*(0.15, 0.01). The intercept was set to −9.0.

### Comparison of statistical methods

We evaluated the power of our *static* stage-wise scale-invariant method in comparison to six other statistical methods. In our main, large-scale analysis using data generated from an enumeration of all possible interaction models (see Material and methods section Generation of synthetic data for estimation of statistical power), we restricted ourselves to statistical methods that could efficiently compute a p-value with enough precision to test how they performed in realistic scenarios: Four methods based on a direct interaction test (i.e., in our framework description above, testing hypothesis *H*_4_ against *H*_*A*_) with a logistic link function GLM, but employing different screening strategies: *Logistic*—no screening. *Marginal+logistic*—the marginal screening method described by [29], which uses a GLM that tests the marginal effect of each variant at an optimistic significance level 0.1 for screening. The screening approaches used in *CSS+logistic* [30] and **R*^2^+logistic* [31] are both *LD*_*cohort*_-based, but differ in the definition of the *χ* ^2^-based statistic, and the choice of significance threshold used for the screening: *χ* ^2^ ≥ 3 (corresponding to *p* ≤ 0.39) and *p* ≤ 10^−4^, respectively. Thirdly, we test two methods based on the *LD*-contrast test with different filtering strategies: **LD*-contrast*—no screening. *Sixpac*—the method of [23], which recodes variant genotypes into two binary variables (according to dominant and recessive coding) and then combines *LD*_*cases*_ screening with a *LD*-contrast main test. The significance level was set to 0.05. We assumed that there were 10^12^ variant pairs present on the chip and that there existed one interacting pair. For the methods without screening (*Logistic* and **LD*-contrast*), as well as for the *Sixpac* method, we corrected for 10^12^ pairs. For the remaining screening methods, we corrected for the expected number of null variant pairs passing the screening, by taking the product of the p-value threshold and the total number of pairs (i.e., $\text{Marginal}+\mathrm{logistic}:\;(\underset{2}{0.1\cdot 10^{6}})\approx 5\cdot 10^{9}$, *CSS+logistic*: 0.39 ⋅ 10^12^ = 3.9 ⋅ 10^11^, and **R*^2^+logistic*: 10^−4^ ⋅ 10^12^ = 10^8^). A pair was declared significant if it passed the significance level of both the screening and the main test. For all these methods we used the Holm-Bonferroni correction for multiple testing, which is more powerful than the classic Bonferroni correction. For the *Static* stage-wise method we corrected for 10^12^ pairs, 100 ⋅ 10^6^ pairs, 100 ⋅ 10^6^ pairs and 4950 pairs in each of the four stages respectively, to simulate the situation with 100 associated variants.

We also performed a second, smaller-scaled, but computationally more demanding, power comparison using data generated from specific interaction models and null models (described in detail in S2 Text). In addition to the seven methods enumerated above, this comparison also included our *adaptive* stage-wise, scale-invariant method and the Model-Based MDR (*MB-MDR*) method [26], which is a parametric extension of the MDR method that addresses some shortcomings of the original MDR method, in particular adjustment for main effects (these methods require the generation of data sets complete with both null and interaction pairs and could not be evaluated in the main power comparison above).

Lastly, we also used the same nine methods in a FWER comparison using simulated data generated as described in Material and methods section Generation of synthetic data for FWER estimation.

### Software availability

A C++ implementation of all methods and source code for all experiments is available at: https://github.com/mfranberg/besiq.

## Supporting Information

S1 Text Proofs. Mathematical proofs for the FWER control of the *static* and *adaptive* method and the proof that the likelihood of models *H*_1_, *H*_2_, *H*_3_ and *H*_*A*_ is invariant of the link function.
(PDF)

S2 Text Supplemental simulations, analyses, and methods. Supplemental power-analyses using simulated data generated from specific interaction models and from null models (shown in S1–S4 Figs). Parameter information for all simulations for power analyses (cf., e.g., S7–S8 Figs). Methods used in the investigation of the impact of weights in the stage-wise methodology (S2 Table). Implementation details of published methods for detection of interaction, used in all power and FWER comparisons.
(PDF)

S1 Fig Statistical power as a function of sample size and minor allele frequency under the double dominant model. The x-axis is the effect level (i.e., categorized effect sizes, cf. S2 Text), and the y-axis is the power. The columns correspond to different minor allele frequencies. The rows correspond to different sample sizes under a balanced design e.g. 2000 indicates 2000 cases and 2000 controls. (Notice that the red line for the *adaptive* method is often hidden behind the black line for the *static* method.)
(TIF)

S2 Fig Statistical power as a function of sample size and minor allele frequency under the double recessive model. The x-axis is the effect level (i.e., categorized effect sizes, cf. S2 Text), and the y-axis is the power. The columns correspond to different minor allele frequencies. The rows correspond to different sample sizes under a balanced design e.g. 2000 indicates 2000 cases and 2000 controls. (Notice that the red line for the *adaptive* method is often hidden behind the black line for the *static* method.)
(TIF)

S3 Fig Statistical power as a function of sample size and minor allele frequency under the XOR model. The x-axis is the effect level (i.e., categorized effect sizes, cf. S2 Text), and the y-axis is the power. The columns correspond to different minor allele frequencies. The rows correspond to different sample sizes under a balanced design e.g. 2000 indicates 2000 cases and 2000 controls. (Notice that the red line for the *adaptive* method is often hidden behind the black line for the *static* method.)
(TIF)

S4 Fig Statistical power as a function of sample size and minor allele frequency under the side model. The x-axis is the effect level (i.e., categorized effect sizes, cf. S2 Text), and the y-axis is the power. The columns correspond to different minor allele frequencies. The rows correspond to different sample sizes under a balanced design e.g. 2000 indicates 2000 cases and 2000 controls.
(TIF)

S5 Fig Estimated penetrances for the rs602633 (PSRC1) and rs4694178 (IL8, CXCL6) interaction in the discovery cohort. The x-axis is the number of minor alleles of the first variant and the y-axis is the estimated penetrance under the different models. The colors correspond to the number of minor alleles of the second variant. The error bars indicate the estimated confidence interval.
(EPS)

S6 Fig Estimated penetrances for the rs602633 (PSRC1) and rs4694178 (IL8, CXCL6) interaction in the replication cohort. The x-axis is the number of minor alleles of the first variant and the y-axis is the estimated penetrance under the different models. The colors correspond to the number of minor alleles of the second variant. The error bars indicate the estimated confidence interval.
(EPS)

S7 Fig The estimated density of effect sizes for models evaluated in the main power experiment represented on the logit scale. The rows correspond to sample sizes, and the columns to heritabilities. The differently colored lines correspond to the estimated density of different parameters in the models. The label “a” refers to the intercept *α*, “b1” and “b2” to the main effects of the first variant *β*_1_ and *β*_2_, “c1” and “c2” to the main effects of the second variant *γ*_1_ and *γ*_2_, and “d11”, “d12”, “d21” and “d22” to the interaction effects *δ*_11_, *δ*_12_, *δ*_21_ and *δ*_22_.
(TIF)

S8 Fig The estimated density of effect sizes for models evaluated in the main power experiment represented on the penetrance scale. The rows correspond to sample sizes, and the columns to heritabilities. The differently colored lines correspond to the estimated density of different parameters in the models. The label “a” refers to the intercept *α*, “b1” and “b2” to the main effects of the first variant *β*_1_ and *β*_2_, “c1” and “c2” to the main effects of the second variant *γ*_1_ and *γ*_2_, and “d11”, “d12”, “d21” and “d22” to the interaction effects *δ*_11_, *δ*_12_, *δ*_21_ and *δ*_22_.
(TIF)

S9 Fig The cumulative distribution function of marginal heritability fraction for the main power experiment. The x-axis represents a threshold for the marginal heritability fraction, and y-axis the fraction models with a marginal heritability fraction greater than this threshold. The blue line represents the empirical cumulative distribution function. The black line represents the cumulative distribution for a uniform distribution. The marginal heritability fraction was computed as the sum of the marginal heritabilities divided by the total heritability.
(TIF)

S1 Table Robustly associated variants genotyped on the IBC chip. A list of the top variants in each of the published CAD loci associated in large-scale meta analyses [2], that are also present on the IBC-chip.
(PDF)

S2 Table Parameters, for the CAD-associated variant pairs, estimated under a logistic regression model. Variant pairs that displayed significant interaction on at least one scale for each strategy in the original analysis of the PROCARDIS cohort are listed. The intercept is *α*. The main effects are *β*_1_, *β*_2_, *γ*_1_, and *γ*_2_. The interaction parameters are *δ*_11_, *δ*_12_, *δ*_21_, and *δ*_22_. LR is the likelihood ratio which measures the degree of evidence for the interaction model compared to the additive model on the logistic scale.
(PDF)

S3 Table Parameters, for the CAD-associated variant pairs, estimated under a logistic regression model after adjustment for population stratification (3 MDS components), age, sex and smoking. Variant pairs that displayed significant interaction on at least one scale for each strategy in the original analysis of the PROCARDIS cohort are listed. The intercept is *α*. The main effects are *β*_1_, *β*_2_, *γ*_1_, and *γ*_2_. The interaction parameters are *δ*_11_, *δ*_12_, *δ*_21_, and *δ*_22_. LR is the likelihood ratio which measures the degree of evidence for the interaction model compared to the additive model on the logistic scale.
(PDF)

S4 Table The genotype frequencies of the significant variant pairs for the discovery and replication analyses. The MAF1 and MAF2 columns denote the minor allele frequency for variant 1 and variant 2 respectively. The counts *n*_*ij*_ is the number of individuals with genotype *i* at the first variant and genotype *j* at the second variant.
(PDF)

S5 Table Evaluation of how different weight combinations affect the statistical power of the *static* and *adaptive* stage-wise methods. The weight for a stage *i* ∈ [4] is indicated by *w*_*i*_. The list is sorted in ascending order by the power obtained for the *static* stage-wise method and the weight combination selected for all remaining analyses in this paper is indicated by bold face.
(PDF)

S6 Table The penetrance matrices used for the double dominant model when comparing the statistical power between stages. The value *p*_*ij*_ denotes the penetrance for genotype *ij*. The rows correspond to the sequence of effect sizes used, sorted in ascending order of the effect size.
(PDF)

S7 Table The penetrance matrices used for the double dominant model when investigating the statistical power for the static method. The value *p*_*ij*_ denotes the penetrance for genotype *ij*. The rows correspond to the sequence of effect sizes used, sorted in ascending order of the effect size.
(PDF)

S8 Table Parameters used for the generation of simulated data from seven different GLMs used to estimate FWER. The GLMS corresponds to the null models used in the stage-wise methodology. The intercept is *α*. The main effects are *β*_1_, *β*_2_, *γ*_1_, and *γ*_2_.
(PDF)
