# Supplementary material for: Discovering Genetic Interactions in Large-Scale Association Studies by Stage-wise Likelihood Ratio Tests
Source: PLoS Genet. 2015 Sep 24;11(9):e1005502. doi: 10.1371/journal.pgen.1005502 (PMC4581725; doi:10.1371/journal.pgen.1005502)
Supplement: S1 Text — Mathematical proofs for the FWER control of the static and adaptive method and the proof that the likelihood of models H 1, H 2, H 3 and H A is invariant of the link function. (PDF) [file pgen.1005502.s001.pdf]

Supporting information S1 Text
for
Discovering genetic interactions in large-scale association studies by stage-wise likelihood ratio tests

Mattias Frånberg, Karl Gertow, Anders Hamsten, Jens Lagergren, Bengt Sennblad

June 22, 2015

1 Proofs that the static and adaptive methods control the family-wise error rate, FWER

1.1 Models and likelihoods

We are investigating a case-control cohort of n subjects. Let $Y \in \{0,1\}^n$ be a binary random variable representing a case-control phenotype, such that $Y_i = 1, i \in [n]$ indicates that a subject i is diseased and $Y_i = 0$ indicates that it is healthy. Let $X_{ij} \in \{0,1,2\}$ be a random variable representing the genotype of variant i in subject j. We will use lower case letters (e.g., x_{ij}) to indicate corresponding (observed) values of these random variables (e.g., X_{ij}).

We are interested in modelling the probability

$$\Pr\left[Y_i = 1 \mid X_{1i} = a, X_{2i} = b\right] = p_{a,b}$$

of being a case given the genotypes for a variant pair (X_1, X_2) and a parameter $p = \{p_{a,b} : a, b \in \{0,1,2\}\}$ and test various assumptions about the parameters of this model. The likelihood for a given pair is then

$$l(p; y \mid x_1, x_2) = \prod_{i=1}^{n} p_{x_{1i},x_{2i}}^{y_i}(1 - p_{x_{1i},x_{2i}})^{1-y_i},$$

There are several possible models for p_{ab} each with a different number of parameters

- $g(p_{a,b}) = \alpha$
- $g(p_{a,b}) = \alpha + \beta_a$
- $g(p_{a,b}) = \alpha + \gamma_b$
- $g(p_{a,b}) = \alpha + \beta_a + \gamma_b$

where g is an invertible function usually called a link function. We enumerate these *null* hypotheses $H_1, \ldots, H_4$ according to the order above. We let H_A be the saturated alternative hypothesis

$$g(p_{a,b}) = \alpha + \beta_a + \gamma_b + \delta_{ab}$$

(notice that for all the models above, we set, where relevant, $\beta_0 = \gamma_0 = \delta_{0b} = \delta_{a0} = 0$ to avoid over-parametrization.)

The hypothesis of interaction states that there is at least one allele pair (a, b) such that $\delta_{ab} \neq 0$. Each of these null hypotheses $H_k, k \in [4]$, may be tested by means of a likelihood ratio statistic

$$\Lambda_k = -2\log\left(\frac{\max_{p\in H_k} l(p; y \mid x_1, x_2)}{\max_{p\in H_A} l(p; y \mid x_1, x_2)}\right) \xrightarrow{d} \chi^2(d_k) \text{ as } n \to \infty,$$

that converges in distribution to a χ^2 random variable with d_k degrees of freedom if hypothesis H_k is true, and

$$\Lambda_k \to \infty$$

if hypothesis H_k is false. The degrees of freedom d_k is the difference in the number of parameters between the saturated model and hypothesis k.

So far we have only considered a single pair, now we must extend the notation to handle multiple pairs. To avoid having more than two indices per variable, we will enumerate the pairs by a single index from 1 to M. We extend our notation in the natural way, so that we have likelihood ratio statistics $\Lambda_{i,k}$, for pair i and hypothesis H_k. We next create a new variable

$$U_{i,k} = 1 - F_k(\Lambda_{i,k}) \xrightarrow{d} U(0,1)$$

where F_k is the χ^2 distribution function with d_k degrees of freedom, the convergence is due to the continuous mapping theorem [1]. This variable corresponds to the p-value for the test statistic. This serves to illustrate that all variables are computed before we determine their significance. We define a per stage test variable, $\phi_{i,k}$, that indicates whether a certain hypothesis was rejected

$$\phi_{i,k} = I(U_{i,k} < \alpha_k)$$

where α_k are different for the static and adaptive method. Finally, we define a joint test variable for the joint stage-wise test up to stage k,

$$\psi_{i,k} = \prod_{l=1}^{k} \phi_{il}.$$

1.2 Proof for the control of FWER

Let $T_i^* \subseteq H$ be a set of indices, such that for each $k \in T_i^*$, H_k is true for variant pair i. We can then define the random variable, E, that counts the number of erroneous rejections as

$$E = \sum_{i=1}^{M} \sum_{k \in T_i^*} \psi_{i,k}.$$

The FWER is now simply the probability of $E > 0$, that is, $\Pr[E > 0]$. We have,

$$\begin{aligned} \Pr[E > 0] &= \Pr\left[\sum_{i=1}^{M} \sum_{k \in T_i^*} \psi_{i,k} > 0\right] \\ &\leq \sum_{i=1}^{M} \Pr\left[\sum_{k \in T_i^*} \psi_{i,k} > 0\right]. \end{aligned} \tag{1}$$

To simplify keeping track of the different T_i^* for different i in the following sections, we will reformulate Equation (1). Let S_τ^* be the set of indices, such that for each $i \in S_\tau^*$, τ is lowest index in T_i^*. Then,

$$\begin{aligned} \Pr[E > 0] &\leq \sum_{i=1}^{M} \Pr\left[\sum_{k \in T_i^*} \psi_{i,k} > 0\right] \\ &= \sum_{\tau=1}^{K} \sum_{i \in S_\tau^*} \Pr\left[\sum_{k \in T_i^*} \psi_{i,k} > 0\right]. \end{aligned} \tag{2}$$

1.2.1 The static method

In this section we will make use of the closed testing principle [2]. This is possible since, for the static method, we have

$$\alpha_k = w_k \frac{\alpha}{V_k},$$

where V_k is an *a priori* estimate of $|S_k^*|$, independent of the variant pair i; such that $V_k \geq |S_k^*|$. Moreover, $H = \{H_1, \ldots, H_K\}$ is closed. That is, H is an ordered set of nested hypothesis such that if $H_k \subseteq H_l$ then $k < l$, and if $T \in [K]$ is a set of indices, then there exists a $\tau \in T$ that constitutes the *intersection hypothesis* of T, that is, $\cap_{k \in T} H_k = H_\tau$. For any arbitrary variant pair i, we have the following Lemma:

Lemma 1. *For a closed set H, suppose that we for each hypothesis $H_k \in H$ have a test $\phi_{i,k}$, such that* $\Pr[\phi_{i,k} = 1 \mid H_k \text{ is true}] = \alpha_k$. *Moreover, let $\psi_{i,k} = \prod_{l=1}^{k} \phi_{i,l}$. Let A be the event that any hypothesis is erroneously rejected for variant pair i, that is, $A = \left\{\sum_{k \in T_i^*} \psi_{i,k} > 0\right\}$, where $T_i^* \subseteq [K]$ contains indices of all true null hypotheses for variant pair i. Then, the event A will be controlled at the level of the intersection hypothesis, H_τ, of T_i^*; in other words:*

$$\Pr[A] \quad = \quad \Pr\left[\sum_{k \in T_i^*} \psi_{i,k} > 0\right] \leq \alpha_\tau.$$

Proof. First, notice that for any $k > 1$, we have $\Pr[\psi_{i,k} > 0] = \Pr[\phi_{i,k} = 1] \Pr[\psi_{i,k-1} > 0 | \phi_{i,k} = 1] \leq \alpha_k$, since $\Pr[\psi_{i,k-1} | \phi_{i,k} = 1] \leq 1$. Let B be the event that $\psi_{i,\tau} = 1$. We have

$$\Pr[A \cap B] = \Pr[B] \Pr[A \mid B] \leq \alpha_\tau$$

since $\Pr[B] = \Pr[\psi_{i,\tau} = 1] \leq \alpha_k$ and $\Pr[A \mid B] \leq 1$. It is clear that $B = \{\psi_{i,\tau} = 1\} \Rightarrow A$. Moreover, since, for any $k \in T_i^*, k > \tau$, we have $\tau \leq k$, it follows, by induction on $\psi_{i,k} = \psi_{i,k-1}\phi_{i,k}$, also that $\neg B = \{\psi_{i,\tau} = 0\} \Rightarrow \{\psi_{i,k} = 0, k \in T_i^*\} = \neg A$. Thus, A occurs if and only if B occurs and, consequently, we have that $A \cap B = A$ and thus $\Pr[A] = \Pr[A \cap B] \leq \alpha_j$ which gives the desired result. This completes the proof. □

We can now state the main results of this section.

Theorem 2. *The static method controls the FWER at the level of α, that is,*

$$\Pr[E > 0] \leq \alpha$$

Proof. Using Equation (2) and Lemma 1, we see that

$$\begin{aligned}
\Pr[E > 0] &\leq \sum_{\tau=1}^{K} \sum_{i \in S_\tau} \Pr\left[\sum_{k \in T_i^*} \psi_{i,k} > 0\right] \\
&\leq \sum_{\tau=1}^{K} \sum_{i \in S_\tau} \alpha_\tau \\
&= \sum_{\tau=1}^{K} \sum_{i \in S_\tau} w_\tau \frac{\alpha}{V_k} \\
&\leq \sum_{\tau=1}^{K} \sum_{i \in S_\tau} w_\tau \frac{\alpha}{|S_\tau|} = \alpha.
\end{aligned}$$

This completes the proof. □

1.2.2 The adaptive method

For the adaptive method, α_k is not fixed *a priori*. Instead, we have

$$\alpha_k = \begin{cases} \frac{w_1\alpha}{K} & \text{if } k = 1 \\ 1 & \text{if } \sum_{i=1}^{K} \phi_{i,k-1} = 0 \\ w_k \frac{\alpha}{\sum_{i=1}^{K} \phi_{i,k-1}} & \text{otherwise.} \end{cases}$$

As a consequence, we cannot rely on the closed testing principle as in Lemma 1. Instead, we must restrict our proof to an asymptotic setting corresponding to a fixed alternative hypothesis. In this case, since $\phi_{i,k}$ is a consistent test, we have that $\phi_{i,k} \xrightarrow{p} 1$ if H_k is false, and consequently, since this imply $\sum_{i=1}^{K} \phi_{i,k-1} \xrightarrow{p} |S_k^*|$, we have

$$\alpha_k = \begin{cases} \frac{w_1\alpha}{K} & \text{if } k = 1 \\ 1 & \text{if } |S_k^*| = 0 \\ w_k \frac{\alpha}{|S_k^*|} & \text{otherwise.} \end{cases}$$

Similarly as for the static method, we start by giving a lemma relating to a fixed arbitrary variant pair i.

Lemma 3. *Let $T_i^* \subseteq H$ be the set of true hypotheses for variant pair i and let τ be the lowest index in T_i^*. Then in the asymptotic case, we have*

$$\sum_{k \in T_i^*} \psi_{i,k} > 0 \Leftrightarrow \phi_{i,\tau} > 0.$$

Proof. We first show that

$$\sum_{k \in T_i^*} \psi_{i,k} > 0 \Leftrightarrow \psi_{i,\tau} > 0. \tag{3}$$

Let τ be the lowest index in T_i^*, then

$$\sum_{k \in T^*} \psi_{i,k} = \psi_{i,\tau} \left(1 + \sum_{k \in T^* \setminus \tau} \prod_{l=\tau}^{k} \phi_{i,k} \right).$$

Since, clearly, $\psi_{i,\tau} = 0 \Leftrightarrow \sum_{k \in T^*} \psi_k = 0$, Equation (3) follows.

Now notice that, in the asymptotic case, $\phi_{i,k} \xrightarrow{p} 1$ if H_k is false. This imply that since H_τ is the first true hypothesis tested, then

$$\psi_{i,\tau} = \phi_{i,\tau} \prod_{k=1}^{\tau-1} \phi_{i,k} = \phi_{i,\tau},$$

which completes the proof.

□

We can now state the main result of this section.

Theorem 4. *In the asymptotic case, the adaptive method controls the FWER at the level of α, that is*

$$\Pr\left[E > 0\right] \leq \alpha.$$

Proof. Using Equation (2) and Lemma 3, we have

$$\begin{aligned}
\Pr[E > 0] &\leq \sum_{\tau=1}^{K} \sum_{i \in S_\tau^*} \Pr\left[\sum_{k \in T_i^*} \psi_{i,k} > 0\right] \\
&= \sum_{\tau=1}^{K} \sum_{i \in S_\tau^*} \Pr[\phi_{i,\tau} > 0] \\
&\leq \sum_{\tau=1}^{K} \sum_{i \in S_\tau^*} \alpha_\tau \\
&= \sum_{\tau=1}^{K} \sum_{i \in S_\tau^*} w_\tau \frac{\alpha}{|S_\tau^*|} = \alpha.
\end{aligned}$$

This completes the proof. □

2 Proof that the likelihood for models H_1, H_2, H_3 and H_A is invariant of the link function.

In this section we will derive closed maximum likelihood expressions for our additive single variant models as well as for the saturated model, and moreover show that the likelihood does not depend on the link function.

We start by considering the additive single variant models

$$\begin{aligned}
g(p_{ab}) &= \alpha, \\
g(p_{ab}) &= \alpha + \beta_a, \text{ and} \\
g(p_{ab}) &= \alpha + \gamma_b.
\end{aligned}$$

Let $n_{i,k}$ be the number of individuals with genotype i and affection k (case/control). Since none of these models depend on both variants we can simplify notation write all models in the form

$$g(p_i) = \alpha + \beta_i \tag{4}$$

substituting β_i for $0, \beta_a$ and γ_b, respectively, to obtain the original models.

Theorem 5. *The maximum likelihood of the model defined by Equation* (4) *is*

$$\max_{\alpha,\beta} l(\alpha, \beta) = \prod_{i=0}^{2} (\frac{n_{i1}}{n_{i1} + n_{i0}})^{n_{i1}} (\frac{n_{i0}}{n_{i1} + n_{i0}})^{n_{i0}},$$

and is consequently independent of the link function $g^{-1}(\alpha, \beta)$.

Proof. The likelihood for this GLM can be written

$$l(\alpha, \beta) = (g^{-1}(\alpha))^{n_{01}} (1 - g^{-1}(\alpha))^{n_{00}} \prod_{i=1}^{2} (g^{-1}(\alpha + \beta_i))^{n_{i1}} (1 - g^{-1}(\alpha + \beta_i))^{n_{i0}}$$

The maximum likelihood score (the gradient of the log likelihood function) equations are

$$\begin{aligned}
\frac{\partial \log l(\alpha, \beta)}{\partial \alpha} &= \frac{n_{01}}{g^{-1}(\alpha)} \frac{\partial g^{-1}(\alpha)}{\partial \alpha} - \frac{n_{00}}{1 - g^{-1}(\alpha)} \frac{\partial g^{-1}(\alpha)}{\partial \alpha} \\
&+ \sum_{i=1}^{2} \frac{n_{i1}}{g^{-1}(\alpha + \beta_i)} \frac{\partial g^{-1}(\alpha + \beta_i)}{\partial \alpha} - \frac{n_{i0}}{1 - g^{-1}(\alpha + \beta_i)} \frac{\partial g^{-1}(\alpha + \beta_i)}{\partial \alpha} = 0 \qquad (5) \\
\frac{\partial \log l(\alpha, \beta)}{\partial \beta_i} &= \frac{n_{i1}}{g^{-1}(\alpha + \beta_i)} \frac{\partial g^{-1}(\alpha + \beta_i)}{\partial \beta_i} - \frac{n_{i0}}{1 - g^{-1}(\alpha + \beta)_i} \frac{\partial g^{-1}(\alpha + \beta_i)}{\partial \beta_i} = 0 \qquad (6)
\end{aligned}$$

We first note that, clearly,

$$\frac{\partial g^{-1}(\alpha+\beta_i)}{\partial \beta_i} = \frac{\partial g^{-1}(\alpha+\beta_i)}{\partial \alpha},$$

and moreover, since g^{-1} is invertible and therefore on-to-one, that

$$\frac{\partial g^{-1}(\alpha+\beta_i)}{\partial \alpha} \neq 0.$$

Thus, simplifying and rearranging Equation (6) gives

$$\begin{aligned} \frac{\partial g^{-1}(\alpha+\beta_i)}{\partial \beta_i}\left(\frac{n_{i1}}{g^{-1}(\alpha+\beta_i)} - \frac{n_{i0}}{1-g^{-1}(\alpha+\beta)_i}\right) &= 0 \\ \frac{n_{i1}}{g^{-1}(\alpha+\beta_i)} - \frac{n_{i0}}{1-g^{-1}(\alpha+\beta)_i} &= 0 \\ g^{-1}(\alpha+\beta_i) &= \frac{n_{i1}}{n_{i1}+n_{i0}} \qquad (7) \end{aligned}$$

Moreover, inserting Equation (6), once for each $i \in \{1,2\}$, into Equation (5) we get

$$\begin{aligned} \frac{n_{01}}{g^{-1}(\alpha)}\frac{\partial g^{-1}(\alpha)}{\partial \alpha} - \frac{n_{00}}{1-g^{-1}(\alpha)}\frac{\partial g^{-1}(\alpha)}{\partial \alpha} &= 0 \\ g^{-1}(\alpha) &= \frac{n_{01}}{n_{01}+n_{00}} \qquad (8) \end{aligned}$$

Using Equations (7) and (8), we get the following likelihood evaluated at the maximum likelihood parameters

$$\max_{\alpha,\beta} l(\alpha,\beta) = (\frac{n_{01}}{n_{01}+n_{00}})^{n_{01}}(\frac{n_{00}}{n_{01}+n_{00}})^{n_{00}}\prod_{i=1}^{2}(\frac{n_{i1}}{n_{i1}+n_{i0}})^{n_{i1}}(\frac{n_{i0}}{n_{i1}+n_{i0}})^{n_{i0}}.$$

Consequently, the likelihood is identical regardless of g. This completes the proof. □

We now consider the saturated model defined by

$$g(p_{ab}) = \alpha + \beta_a + \gamma_b + \delta_{ab}.$$

For this model, we have the following theorem:

Theorem 6. *The maximum likelihood expression for*

$$\hat{l}(\alpha,\beta,\gamma,\delta) = \prod_{i=0}^{2}\prod_{j=0}^{2}(\frac{n_{ij1}}{n_{ij1}+n_{ij0}})^{n_{ij1}}(\frac{n_{ij0}}{n_{ij1}+n_{ij0}})^{n_{ij0}}$$

and is consequently invariant to the choice of link function.

The proof is omitted, but can, in a corresponding way to the proof for Theorem 5 above, be derived by setting up and solving the corresponding system of maximum likelihood score equations.

We end by observing that there are no similar closed form expressions for the additive model $g(p_{ab}) = \alpha + \beta_i + \gamma_j$ that are independent of the choice of link function.

References

[1] Mann HB, Wald A. On stochastic limit and order relationships. The Annals of Mathematical Statistics. 1943;14(3):217–226.

[2] Marcus R, Eric P, Gabriel KR. On Closed Testing Procedures with Special Reference to Ordered Analysis of Variance. Biometrika. 1976 December;63:655–660.
