# Supplementary material for: Discovering Genetic Interactions in Large-Scale Association Studies by Stage-wise Likelihood Ratio Tests
Source: PLoS Genet. 2015 Sep 24;11(9):e1005502. doi: 10.1371/journal.pgen.1005502 (PMC4581725; doi:10.1371/journal.pgen.1005502)
Supplement: S2 Text — Supplemental power-analyses using simulated data generated from specific interaction models and from null models (shown in S1–S4 Figs). Parameter information for all simulations for power analyses (cf., e.g., S7–S8 Figs). Methods used in the investigation of the impact of weights in the stage-wise methodology (S2 Table). Implementation details of published methods for detection of interaction, used in all power and FWER comparisons. (PDF) [file pgen.1005502.s002.pdf]

Supporting information **S2 Text**
for
Discovering genetic interactions in large-scale association studies by stage-wise likelihood ratio tests

Mattias Frånberg, Karl Gertow, Anders Hamsten, Jens Lagergren, Bengt Sennblad

June 22, 2015

Power for specific models of interaction

There are numerous models of interaction that can be tested. In the main text, we evaluate our static stage-wise method and seven additional methods on simulated data generated from a spectrum covering all possible interaction models. Here, we supplement that analysis with a power comparison of nine methods using data simulated from specific interaction models together with data from null models. We evaluated four specific interaction models described in Table S2_text.1. The two first models describe interaction caused by the combination of risk alleles from each variant, either in a dominant manner (*Double dominant*) or in a recessive manner (*Double recessive*). The last two models describe interaction caused by heterozygote-homozygote combinations, either by all possible such combinations (*XOR*) by one specific such combination (*Side*).

We used a similar setup as in the main text Materials and methods section Generation of synthetic data for FWER estimation. That is we constructed data sets that contained both unassociated and associated variants. The genotypes for the unassociated variants were generated according to Hardy-Weinberg equilibrium with a minor allele frequency sampled uniformly between 0.2 and 0.4. Each dataset also contained a single interacting pair. The genotypes of the variants corresponding to the interacting pair were generated according to a minor allele frequency that for both variants were varied over 0.2, 0.3 and 0.4. We assumed a balanced design, and sampled individuals until they totaled the desired sample size of 2000, 3000 and 4000 samples each in the case and control groups. For each combination of model parameters, minor allele frequency and sample size, 200 replicates containing $N = 500$ variants were generated. The parameters of the interaction models, along with resulting penetrances, heritability and marginal heritability can be found in section Simulation parameters and their interpretation as specific GLMs.

We evaluated the statistical power for our *(i) adaptive* and *(ii) static* stage-wise methods and the following seven additional methods: the *(iii) Logistic, (iv) Marginal+logistic, (v) CSS+logistic, (vi)* R^2*+logistic, (vii) LD-contrast, (viii) Sixpac* and *(ix) MB-MDR* methods. The significance level was set to 0.05. For the methods without screening (iii and vii), as well as for the *Sixpac* method, we corrected for $N(N-1)/2 = 124,750$ pairs. For the remaining screening methods, we corrected for the number of variant pairs passing the screening. For *MB-MDR*, a permutation approach was used to determine adjusted p-values. For the static stage-wise method we corrected for $124,750$ pairs, $2N = 1,000$ pairs, $1,000$ pairs and 1 pair in each of the 4 stages respectively. The adaptive stage-wise method determines the multiple correction dynamically.

In the Double dominant model results (S1 Fig), *MB-MDR* and *Sixpac* perform relatively better for low MAF. However, at higher MAFs, the performance of in particular *Sixpac* deteriorates and the *Adaptive* and *Static* stage-wise methods surpasses the other methods in terms of power. The Double recessive model (S2 Fig) appears to be challenging. No method performs well at low

MAF. At intermediate MAF, *Sixpac* clearly performs best, but as the MAF increases further, the *Adaptive* and *Static* stage-wise methods catch up in performance. For high MAF and large sample size, most methods perform well. In the XOR model results (S3 Fig), again the *MB-MDR* and *Sixpac* methods performs best at lower MAFs, and again the performance of *Sixpac* deteriorates at higher MAF. At higher MAFs, our *Adaptive* and *Static* stage-wise methods perform well, but are surpassed by the *CSS+logistic*, *MB-MDR*, and *logistic* methods. Finally, in the Side model results (S4 Fig), *MB-MDR* and *Sixpac* perform relatively better on low MAFS. At higher MAFS, the *Adaptive* and *Static* stage-wise methods clearly surpasses the other methods, while the performance of *Sixpac* deteriorates. For all models, the *LD-contrast* and R^2*+logistic* methods have the relatively worst performance. The R^2*+logistic* method has a very strict p-value threshold at the screening stage that probably contributes to the poor power; a positive effect of this strict threshold is that the R^2*+logistic* method controls the FWER better than the other methods using a logit link function (main text table 2).

In summary, the results is consistent with that from the main text analysis (which was based on data generated from all possible interaction models) with some variation. Our *Static* stage-wise method, together with our *Adaptive* stage-wise method (which was not tested in the main text analysis), consistently performs among the top, while the *LD-contrast* and R^2*+logistic* methods are among the worst. However, we can see that the power of the *Sixpac* method, which performed rather mediocre in the main text power analysis, varies for different MAFs; at low MAF, it performs relatively better than most other methods for all models. However, except for the double recessive model, its performance clearly becomes worse as MAF increases. *MB-MDR* also performs relatively well at low MAF, but, while its performance does not get worse, it takes, together with *logistic*, *CSS+logistic* and often also *marginal+logistic*, an intermediate position in terms of power. The exception is the XOR model, where *MB-MDR*, *logistic* and *CSS+logistic* models perform best.

Table S2_text.1: This table describes 4 specific interaction models. The parameter β_0 can be thought of as the population penetrance when not considering genetic effects, β_1 is the increase in penetrance when having the specific genotype. In the dominant model there is an increased risk when at least one risk allele is present in both loci. In the homozygote model there is an increased risk when both loci are homozygous for the risk allele. In the XOR model there is an increased risk with the *AABB* and *aabb*. In the side model there is an increased risk for the *AABb* genotype.

Double dominant				Double recessive			
Genotype	AA	Aa	aa	Genotype	AA	Aa	aa
BB	β_0	β_0	β_0	BB	β_0	β_0	β_0
Bb	β_0	$\beta_0+\beta_1$	$\beta_0+\beta_1$	Bb	β_0	β_0	β_0
bb	β_0	$\beta_0+\beta_1$	$\beta_0+\beta_1$	bb	β_0	β_0	$\beta_0+\beta_1$
XOR				Side			
Genotype	AA	Aa	aa	Genotype	AA	Aa	aa
BB	β_0	$\beta_0+\beta_1$	β_0	BB	β_0	β_0	β_0
Bb	$\beta_0+\beta_1$	β_0	$\beta_0+\beta_1$	Bb	$\beta_0+\beta_1$	β_0	β_0
bb	β_0	$\beta_0+\beta_1$	β_0	bb	β_0	β_0	β_0

Simulation parameters and their interpretation as specific GLMs

To enhance interpreting our simulated data to specific GLMs, we here provide tables for the simulated data generated from specific interaction models, described above in section Power for specific models of interaction, and corresponding figures for the simulated data generated from all possible interaction model, described in the main text Material and methods section Generation of synthetic data for estimation of statistical power.

Specific interaction model simulations

The penetrance matrices used in the simulations are shown in Table S2_text.2. While we have used the penetrances directly in our simulations, each penetrance matrix can equivalently be expressed as a GLM with any choice of invertible link function. This is accomplished by considering two scales as two equivalent parameterizations of the same model, let $\theta = \{p_{ij}\}_{i,j\in\{0,1,2\}^2}$ be the vector of parameters corresponding to the penetrances and $\beta = \{\alpha, \beta_1, \beta_2, \gamma_1, \gamma_2, \delta_{11}, \delta_{12}, \delta_{21}, \delta_{22}\}$ be the vector of parameters corresponding to the regression model. Let P be the 9×9 matrix that maps β to the effect level for each genotype, we then have the following relationship between θ and β

$$\theta = g^{-1}(P\beta)$$

and thus given θ we can express it in terms of β by

$$\beta = P^{-1}g(\theta)$$

The corresponding regression coefficients for a GLM with an logit and a identity link function are shown in Tables S2_text.3 and S2_text.4, respectively. Notice, however, that the size of the interaction parameters cannot be directly compared between models because their standard errors depend on the link function.

We remark that apart from the intercept variable ('a' in the table headers) and the scale, the relative distributions of regression coefficients are remarkably similar between the equivalent identity and the logit link function GLMs.

To further investigate the properties of the interaction models from which we generate data, we compute the heritability, H^2, for each model, MAF-combination and effect level, and decompose it into marginal heritability and "interaction" heritability (Table S2_text.5). For each variant i in the interacting variant pair, the marginal heritability, H_i^2, is computed from the marginal penetrances in the penetrance matrix. The interaction heritability, H_{12}^2 is then obtained as $H_{12}^2 = H^2 - H_1^2 - H_2^2$. The parameter determining the effect size, β_1, was varied at four different effect levels, determined individually for each of the four generative models to retain a prevalence approximately in the interval $[0.1, 0.2]$ and at the same time obtain an interval that captured the performance of the tested method to infer interaction. We can see that the heritability represented by this interval varies slightly between models. More interestingly, the heritability fractions (H_i^2/H^2 and H_{12}^2/H^2) and in particular the ratio between interaction and marginal heritabilities ($H_{12}^2/(H_1^2 + H_2^2)$) shows that there is a substantial difference in the relative amount of heritability that can be attributed to interaction. For the *Double dominant* and *Side* models, this amount is rather small, the interaction–marginal heritability ratio lies between $0.28 - 0.89$, while for the *Double recessive* and *XOR* models, it is much larger, the ratio here lies between $2.6 - 312$. Interestingly, for the *Double dominant* and *Double recessive* model, the relative interaction heritability decreases with MAF, while for the *XOR* and *Side* models it increases. In summary, we suggest that this indicates that our choice of specific models captures a reasonable section of the variability of interaction models.

Main text simulations from the spectrum of all possible interaction models

Tabulation of the penetrance matrices and equivalent GLMs for the large-scale simulation data generated from all possible interaction models in the main text would be impractical. Instead, we plot the density distributions, over all generated models, of the regression coefficients from the logit (S7 Fig) and identity (S8 Fig) link function GLMs that are equivalent to the penetrance matrices, for each combination of sample size and MAF. In both the logit and the identity figure, the main and interaction effects are, for the majority of models, concentrated around the same effect sizes. The cumulative marginal heritability fraction in S9 Fig shows that the models cover the range from small to large interaction heritability in a fairly even manner. As many, if not most, interaction models induce a marginal effect, there is a slight bias towards models with a larger marginal heritability, around 71% of the models have a marginal heritability fraction greater than 50%.

Table S2_text.2: Penetrance matrices from the simulations using specific interaction models, listing penetrances p_{ij} for each allele combination ij.

Model	p_{00}	p_{01}	p_{02}	p_{10}	p_{11}	p_{12}	p_{20}	p_{21}	p_{22}
Double dominant	0.1000	0.1000	0.1000	0.1000	0.1200	0.1200	0.1000	0.1200	0.1200
Double dominant	0.1000	0.1000	0.1000	0.1000	0.1500	0.1500	0.1000	0.1500	0.1500
Double dominant	0.1000	0.1000	0.1000	0.1000	0.1800	0.1800	0.1000	0.1800	0.1800
Double dominant	0.1000	0.1000	0.1000	0.1000	0.2100	0.2100	0.1000	0.2100	0.2100
Double recessive	0.1000	0.1000	0.1000	0.1000	0.1000	0.1000	0.1000	0.1000	0.2000
Double recessive	0.1000	0.1000	0.1000	0.1000	0.1000	0.1000	0.1000	0.1000	0.2500
Double recessive	0.1000	0.1000	0.1000	0.1000	0.1000	0.1000	0.1000	0.1000	0.3000
Double recessive	0.1000	0.1000	0.1000	0.1000	0.1000	0.1000	0.1000	0.1000	0.3500
XOR	0.1000	0.1200	0.1000	0.1200	0.1000	0.1200	0.1000	0.1200	0.1000
XOR	0.1000	0.1400	0.1000	0.1400	0.1000	0.1400	0.1000	0.1400	0.1000
XOR	0.1000	0.1600	0.1000	0.1600	0.1000	0.1600	0.1000	0.1600	0.1000
XOR	0.1000	0.1800	0.1000	0.1800	0.1000	0.1800	0.1000	0.1800	0.1000
Side	0.1000	0.1000	0.1000	0.1200	0.1000	0.1000	0.1000	0.1000	0.1000
Side	0.1000	0.1000	0.1000	0.1500	0.1000	0.1000	0.1000	0.1000	0.1000
Side	0.1000	0.1000	0.1000	0.1800	0.1000	0.1000	0.1000	0.1000	0.1000
Side	0.1000	0.1000	0.1000	0.2100	0.1000	0.1000	0.1000	0.1000	0.1000

Table S2_text.3: This table shows the regression coefficients for each parameter combination on the logit scale.

Model	α	β_1	β_2	γ_1	γ_2	δ_{11}	δ_{12}	δ_{21}	δ_{22}
Double dominant	-2.1972	0.0000	0.0000	0.0000	0.0000	0.2048	0.2048	0.2048	0.2048
Double dominant	-2.1972	0.0000	0.0000	0.0000	0.0000	0.4626	0.4626	0.4626	0.4626
Double dominant	-2.1972	0.0000	0.0000	0.0000	0.0000	0.6809	0.6809	0.6809	0.6809
Double dominant	-2.1972	0.0000	0.0000	0.0000	0.0000	0.8723	0.8723	0.8723	0.8723
Double recessive	-2.1972	0.0000	0.0000	0.0000	0.0000	0.0000	0.0000	0.0000	0.8109
Double recessive	-2.1972	0.0000	0.0000	0.0000	0.0000	0.0000	0.0000	0.0000	1.0986
Double recessive	-2.1972	0.0000	0.0000	0.0000	0.0000	0.0000	0.0000	0.0000	1.3499
Double recessive	-2.1972	0.0000	0.0000	0.0000	0.0000	0.0000	0.0000	0.0000	1.5782
XOR	-2.1972	0.2048	0.0000	0.2048	0.0000	-0.4096	0.0000	0.0000	0.0000
XOR	-2.1972	0.3819	0.0000	0.3819	0.0000	-0.7639	0.0000	0.0000	0.0000
XOR	-2.1972	0.5390	0.0000	0.5390	0.0000	-1.0780	0.0000	0.0000	0.0000
XOR	-2.1972	0.6809	0.0000	0.6809	0.0000	-1.3618	0.0000	0.0000	0.0000
Side	-2.1972	0.0000	0.0000	0.2048	0.0000	-0.2048	-0.2048	0.0000	0.0000
Side	-2.1972	0.0000	0.0000	0.4626	0.0000	-0.4626	-0.4626	0.0000	0.0000
Side	-2.1972	0.0000	0.0000	0.6809	0.0000	-0.6809	-0.6809	0.0000	0.0000
Side	-2.1972	0.0000	0.0000	0.8723	0.0000	-0.8723	-0.8723	0.0000	0.0000

Table S2_text.4: This table shows the regression coefficients for each parameter combination on the penetrance scale.

Model	α	β_1	β_2	γ_1	γ_2	δ_{11}	δ_{12}	δ_{21}	δ_{22}
Double dominant	0.1000	0.0000	0.0000	0.0000	0.0000	0.0200	0.0200	0.0200	0.0200
Double dominant	0.1000	0.0000	0.0000	0.0000	0.0000	0.0500	0.0500	0.0500	0.0500
Double dominant	0.1000	0.0000	0.0000	0.0000	0.0000	0.0800	0.0800	0.0800	0.0800
Double dominant	0.1000	0.0000	0.0000	0.0000	0.0000	0.1100	0.1100	0.1100	0.1100
Double recessive	0.1000	0.0000	0.0000	0.0000	0.0000	0.0000	0.0000	0.0000	0.1000
Double recessive	0.1000	0.0000	0.0000	0.0000	0.0000	0.0000	0.0000	0.0000	0.1500
Double recessive	0.1000	0.0000	0.0000	0.0000	0.0000	0.0000	0.0000	0.0000	0.2000
Double recessive	0.1000	0.0000	0.0000	0.0000	0.0000	0.0000	0.0000	0.0000	0.2500
XOR	0.1000	0.0200	0.0000	0.0200	0.0000	-0.0400	0.0000	0.0000	0.0000
XOR	0.1000	0.0400	0.0000	0.0400	0.0000	-0.0800	0.0000	0.0000	0.0000
XOR	0.1000	0.0600	0.0000	0.0600	0.0000	-0.1200	0.0000	0.0000	0.0000
XOR	0.1000	0.0800	0.0000	0.0800	0.0000	-0.1600	0.0000	0.0000	0.0000
Side	0.1000	0.0000	0.0000	0.0200	0.0000	-0.0200	-0.0200	0.0000	0.0000
Side	0.1000	0.0000	0.0000	0.0500	0.0000	-0.0500	-0.0500	0.0000	0.0000
Side	0.1000	0.0000	0.0000	0.0800	0.0000	-0.0800	-0.0800	0.0000	0.0000
Side	0.1000	0.0000	0.0000	0.1100	0.0000	-0.1100	-0.1100	0.0000	0.0000

The effect of different weights in the stage-wise analysis

The stage-wise method requires weights to be selected. We performed a simple experiment to evaluate the impact of the weight selection on statistical power. In this experiment genotypes were taken from chromosome 20 genotyped on the IBC-chip in the PROCARDIS cohort. We investigated power for all non-zero weight combinations that summed to 1 with a precision of 0.1. For each specific weight combination, we generated 1000 random penetrance matrices. For each penetrance matrix, a random variant pair was selected and the phenotype was then generated according to the penetrance matrix. The penetrance for each genotype combination was sampled from the normal distribution $\mathrm{N}(\beta_0, \sqrt{H^2\beta_0(1-\beta_0)})$ (penetrances were truncated to $(0.1, 0.9)$), where H^2 is the given heritability (as defined in the main text) and β_0 is the population risk. The H_2 was set to 0.02 and β_0 to 0.5.

We ran both the *static* and *adaptive* method, and computed the statistical power for each weight combination over the 1000 replicates. The general conclusion of this experiment is that for these weight combinations there is only a small difference in power, and in this analysis the weight combination 0.1, 0.3, 0.3 and 0.3 performed best. This weight combination was used in all other stage-wise analyses in this paper.

Implementation details

For several of the methods an no implementation was available for large-scale analyses on Unix clusters. Here we describe how these methods were implemented and what assumptions were made. Let N_{ij} be the observed number of counts for the combination of genotypes i for the first variant and j for the second variant.

Logistic main test

The *logistic* main test was implemented as a GLM with a logit link function (described in the main text section Interaction in the generalized linear model). Of note, we did not assume additivity. The implementation of the various screening tests is described below. The Holm-Bonferroni method was used for multiple test correction; when no screening was applied we corrected for all

Table S2_text.5: Heritabilities and prevalence for simulated data under different minor allele frequencies (MAFs) and effect level. H^2 denotes the heritability, H_1^2 and H_2^2 denotes the marginal heritabilities, and H_{12}^2 denotes the heritability attributable to interaction.

Model	MAF1	MAF2	Effect level	H^2	$\frac{H_1^2}{H^2}$	$\frac{H_2^2}{H^2}$	$\frac{H_{12}^2}{H^2}$	$\frac{H_{12}^2}{H_1^2+H_2^2}$	Prevalence
Double dominant	0.2000	0.2000	0	0.0005	0.2647	0.2647	0.4706	0.8889	0.1026
Double dominant	0.2000	0.2000	1	0.0030	0.2647	0.2647	0.4706	0.8889	0.1065
Double dominant	0.2000	0.2000	2	0.0074	0.2647	0.2647	0.4706	0.8889	0.1104
Double dominant	0.2000	0.2000	3	0.0135	0.2647	0.2647	0.4706	0.8889	0.1143
Double dominant	0.3000	0.3000	0	0.0008	0.3377	0.3377	0.3245	0.4804	0.1052
Double dominant	0.3000	0.3000	1	0.0048	0.3377	0.3377	0.3245	0.4804	0.1130
Double dominant	0.3000	0.3000	2	0.0116	0.3377	0.3377	0.3245	0.4804	0.1208
Double dominant	0.3000	0.3000	3	0.0208	0.3377	0.3377	0.3245	0.4804	0.1286
Double dominant	0.4000	0.4000	0	0.0010	0.3902	0.3902	0.2195	0.2813	0.1082
Double dominant	0.4000	0.4000	1	0.0057	0.3902	0.3902	0.2195	0.2813	0.1205
Double dominant	0.4000	0.4000	2	0.0134	0.3902	0.3902	0.2195	0.2812	0.1328
Double dominant	0.4000	0.4000	3	0.0236	0.3902	0.3902	0.2195	0.2812	0.1451
Double recessive	0.2000	0.2000	0	0.0002	0.0385	0.0385	0.9231	12.0000	0.1002
Double recessive	0.2000	0.2000	1	0.0004	0.0385	0.0385	0.9231	12.0000	0.1002
Double recessive	0.2000	0.2000	2	0.0007	0.0385	0.0385	0.9231	12.0000	0.1003
Double recessive	0.2000	0.2000	3	0.0011	0.0385	0.0385	0.9231	12.0000	0.1004
Double recessive	0.3000	0.3000	0	0.0009	0.0826	0.0826	0.8349	5.0556	0.1008
Double recessive	0.3000	0.3000	1	0.0020	0.0826	0.0826	0.8349	5.0556	0.1012
Double recessive	0.3000	0.3000	2	0.0035	0.0826	0.0826	0.8349	5.0556	0.1016
Double recessive	0.3000	0.3000	3	0.0055	0.0826	0.0826	0.8349	5.0556	0.1020
Double recessive	0.4000	0.4000	0	0.0027	0.1379	0.1379	0.7241	2.6250	0.1026
Double recessive	0.4000	0.4000	1	0.0060	0.1379	0.1379	0.7241	2.6250	0.1038
Double recessive	0.4000	0.4000	2	0.0106	0.1379	0.1379	0.7241	2.6250	0.1051
Double recessive	0.4000	0.4000	3	0.0164	0.1379	0.1379	0.7241	2.6250	0.1064
XOR	0.2000	0.2000	0	0.0010	0.1147	0.1147	0.7705	3.3580	0.1087
XOR	0.2000	0.2000	1	0.0038	0.1147	0.1147	0.7705	3.3580	0.1174
XOR	0.2000	0.2000	2	0.0080	0.1147	0.1147	0.7705	3.3580	0.1261
XOR	0.2000	0.2000	3	0.0135	0.1147	0.1147	0.7705	3.3580	0.1348
XOR	0.3000	0.3000	0	0.0010	0.0250	0.0250	0.9501	19.0312	0.1097
XOR	0.3000	0.3000	1	0.0038	0.0250	0.0250	0.9501	19.0312	0.1195
XOR	0.3000	0.3000	2	0.0080	0.0250	0.0250	0.9501	19.0313	0.1292
XOR	0.3000	0.3000	3	0.0134	0.0250	0.0250	0.9501	19.0313	0.1390
XOR	0.4000	0.4000	0	0.0010	0.0016	0.0016	0.9968	312.0000	0.1100
XOR	0.4000	0.4000	1	0.0038	0.0016	0.0016	0.9968	312.0000	0.1200
XOR	0.4000	0.4000	2	0.0080	0.0016	0.0016	0.9968	312.0000	0.1300
XOR	0.4000	0.4000	3	0.0133	0.0016	0.0016	0.9968	312.0000	0.1399
Side	0.2000	0.2000	0	0.0007	0.5473	0.1449	0.3078	0.4448	0.1041
Side	0.2000	0.2000	1	0.0042	0.5473	0.1449	0.3078	0.4448	0.1102
Side	0.2000	0.2000	2	0.0101	0.5473	0.1449	0.3078	0.4448	0.1164
Side	0.2000	0.2000	3	0.0183	0.5473	0.1449	0.3078	0.4448	0.1225
Side	0.3000	0.3000	0	0.0007	0.3578	0.2697	0.3725	0.5935	0.1041
Side	0.3000	0.3000	1	0.0042	0.3578	0.2697	0.3725	0.5935	0.1103
Side	0.3000	0.3000	2	0.0102	0.3578	0.2697	0.3725	0.5935	0.1165
Side	0.3000	0.3000	3	0.0184	0.3578	0.2697	0.3725	0.5935	0.1226
Side	0.4000	0.4000	0	0.0006	0.2263	0.3714	0.4023	0.6731	0.1035
Side	0.4000	0.4000	1	0.0037	0.2263	0.3714	0.4023	0.6731	0.1086
Side	0.4000	0.4000	2	0.0091	0.2263	0.3714	0.4023	0.6731	0.1138
Side	0.4000	0.4000	3	0.0165	0.2263	0.3714	0.4023	0.6731	0.1190

variant pairs tested, while when screening was applied we corrected for expected number of pairs passing the screening.

Marginal screening

We used a logit-link-function GLM with 3 parameters to test the marginal effect of each variant, using a 2 degrees of freedom test. We declared all marginal effects with a p-value less than 0.1 significant. For the main test, all possible pairs that contain at least one significant marginal effect was tested using a logistic regression model using a 4 degrees of freedom test.

CSS screening

Let $g_i = \sum_{j+k=i} N_{ij}$ and, for convenience of notation, define $g_3 = E[g_3] = 0$. Then the CSS χ^2-statistic of [?] can be written

$$CSS = \sum_{i=0}^{4} \frac{g_i - E[g_i]}{E[g_i]}.$$

The expected value $E[g_i]$ was estimated using the marginal frequencies,

$$E[g_i] = \sum_{j+k=3} \frac{N_{\cdot j} N_{k\cdot}}{N}.$$

We did not implement the technique of reducing the degrees of freedom if a marginal effect was not significant. We used a threshold of $CSS \geq 3$ in the first stage as recommended in the paper, this corresponds to a p-value of 0.39.

R^2 screening

The test described in [1] included an erroneous expression for the variance that made the test overly conservative. In our implementation, we used the expression from [2] amended with an addition factor N. Let u_i and v_j be the weights for these genotypes, as defined by [1]. We have the test statistic

$$T = \sum_{ij} u_i v_j N_{ij},$$

its mean under the null model

$$E[T] = \sum_{ij} u_i v_j \frac{N_{\cdot j} N_{i\cdot}}{N},$$

and its variance under the null model

$$Var[T] = \frac{\left(\sum_i u_i^2 N_{i\cdot} - (\sum_i u_i N_{i\cdot})^2/N\right)\left(\sum_j v_j^2 N_{\cdot j} - (\sum_j u_j N_{\cdot j})^2/N\right)}{N}.$$

The Wald statistic W was then computed by

$$W = \frac{(T - E[T])^2}{Var[T]}$$

and compared to a $\chi^2(1)$ distribution. We assumed an additive model for the weights so that $u = v = (0, 1, 2)$. We used a significance threshold of 10^{-4} in the first stage. The second stage used a 4 degrees of freedom interaction test in a logistic regression model.

LD-contrast main test

For each subcohort $set \in \{cases, controls\}$, let p and q be the minor allele frequency of the first and second variant in the population, respectively, and let p_{00} be the population frequency of the haplotype that contains both minor alleles. Let $n = |set|$, x_{ij} be the observed frequency of genotype in set, and let $\hat{p} = \sum_{i=0}^{2} x_{0i}$ and $\hat{q} = \sum_{i=0}^{2} x_{i0}$. The difference between the haplotype frequency and the product of the marginal frequencies $D = p_{00} - pq$ was estimated in both the case and control population using

$$\hat{D}_{set} = \frac{1}{2(n-1)} \sum_{i=1}^{n} (x_{i1} - 2\hat{p})(x_{i2} - 2\hat{q})$$

using the fact that D is twice the covariance between the two variants. The differences were then compared between cases and controls using the following test

$$\frac{(\hat{D}_{cases} - \hat{D}_{controls})^2}{Var[\hat{D}_{cases} - \hat{D}_{controls}]}$$

where

$$Var[\hat{D}_{cases} - \hat{D}_{controls}] = \frac{\hat{p}_{cases}(1-\hat{p}_{cases})\hat{q}_{cases}(1-\hat{q}_{cases})}{n_{cases}} + \frac{\hat{p}_{controls}(1-\hat{p}_{controls})\hat{q}_{controls}(1-\hat{q}_{controls})}{n_{controls}}.$$

We applied Holm-Bonferroni correction for all variant pairs tested.

Sixpac and MB-MDR

For the *Sixpac* and *MB-MDR* approaches, we used the Unix-distribution made available by the authors (`http://www.cs.columbia.edu/~snehitp/sixpac/` and `http://www.statgen.ulg.ac.be/software_mbmdr.html`, respectively). For *Sixpac*, we applied Holm-Bonferroni correction for all variant pairs tested (as recommended by the authors), and for *MB-MDR*, we used the adjusted p-values from the program, and specified to perform permutation for the top 10 interaction pairs.

References

[1] Lewinger JP, Morrison JL, Thomas DC, Murcray CE, Conti DV, Li D, et al. Efficient Two-Step Testing of Gene-Gene Interactions in Genome-Wide Association Studies. Genet Epidemiol. 2013 April;37(5):440–451.

[2] Agresti A, Mehta CR, Patel NR. Exact Inference for Contingency Tables with Ordered Categories. Journal of the American Statistical Association. 1990;85(410):453–458.
