# Supplementary figures and images for: Discovering Genetic Interactions in Large-Scale Association Studies by Stage-wise Likelihood Ratio Tests
Source: PLoS Genet. 2015 Sep 24;11(9):e1005502. doi: 10.1371/journal.pgen.1005502 (PMC4581725; doi:10.1371/journal.pgen.1005502)

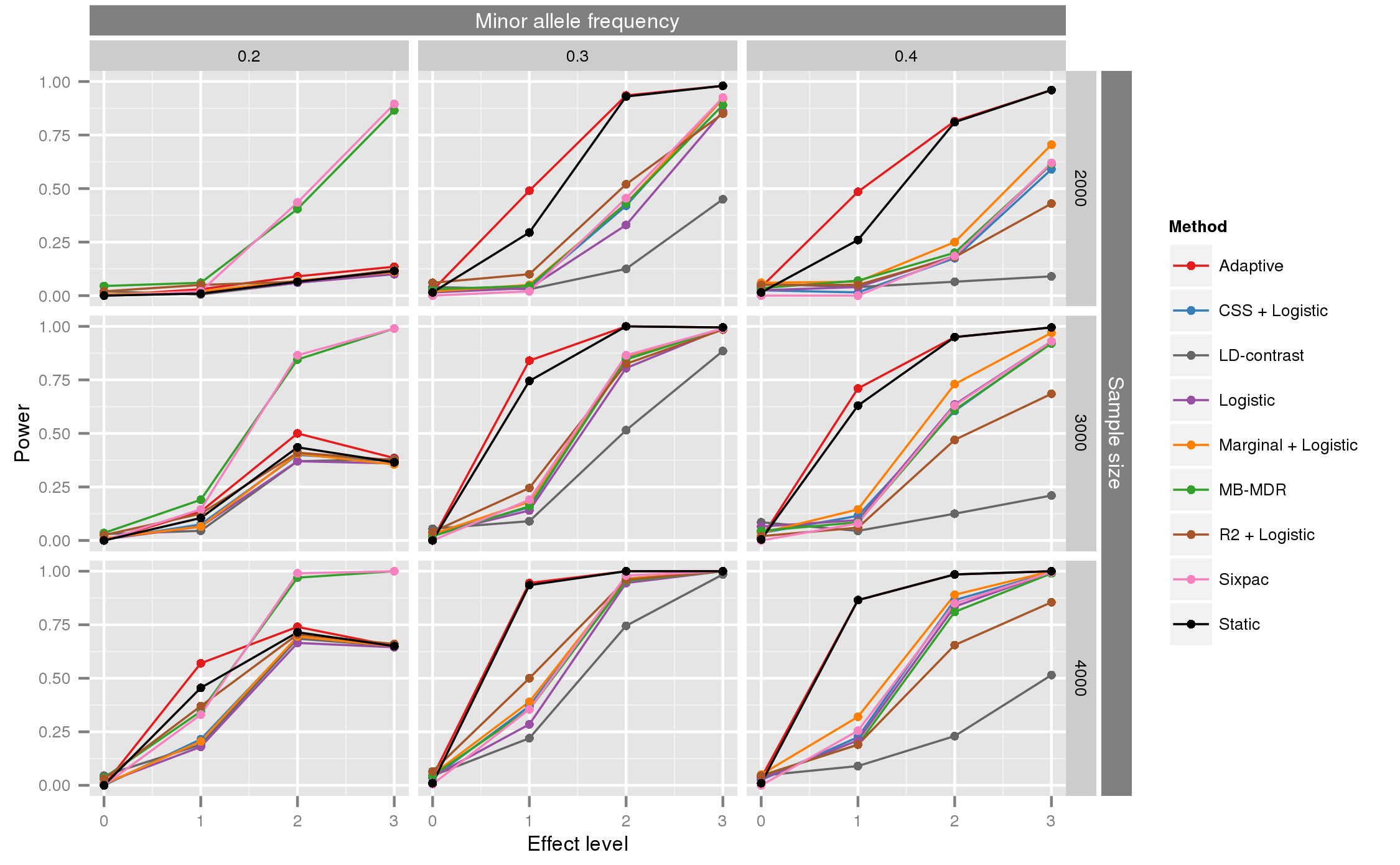

Supplement: S1 Fig — The x-axis is the effect level (i.e., categorized effect sizes, cf. S2 Text), and the y-axis is the power. The columns correspond to different minor allele frequencies. The rows correspond to different sample sizes under a balanced design e.g. 2000 indicates 2000 cases and 2000 controls. (Notice that the red line for the adaptive method is often hidden behind the black line for the static method.) (TIF) [file pgen.1005502.s003.tif]

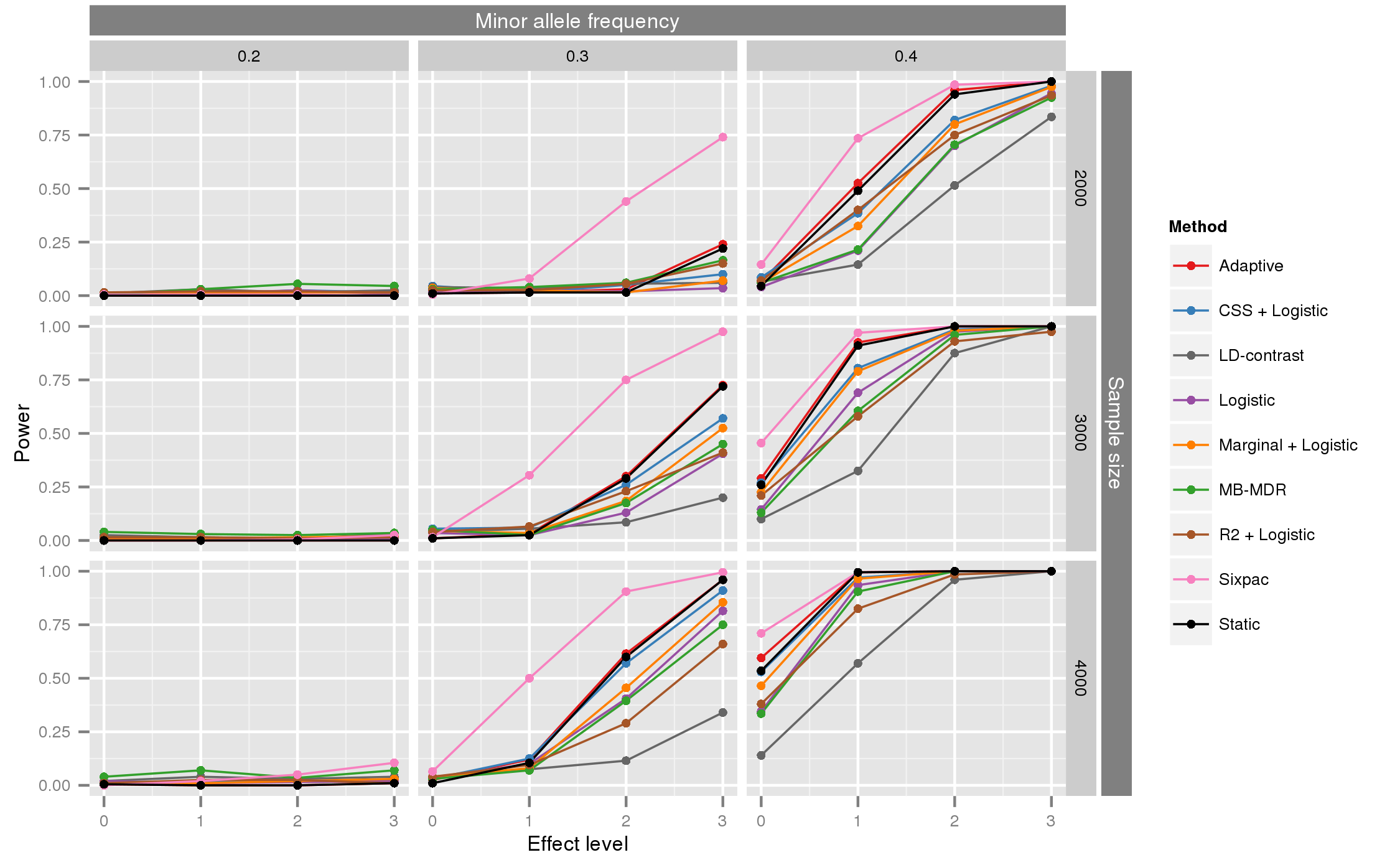

Supplement: S2 Fig — The x-axis is the effect level (i.e., categorized effect sizes, cf. S2 Text), and the y-axis is the power. The columns correspond to different minor allele frequencies. The rows correspond to different sample sizes under a balanced design e.g. 2000 indicates 2000 cases and 2000 controls. (Notice that the red line for the adaptive method is often hidden behind the black line for the static method.) (TIF) [file pgen.1005502.s004.tif]

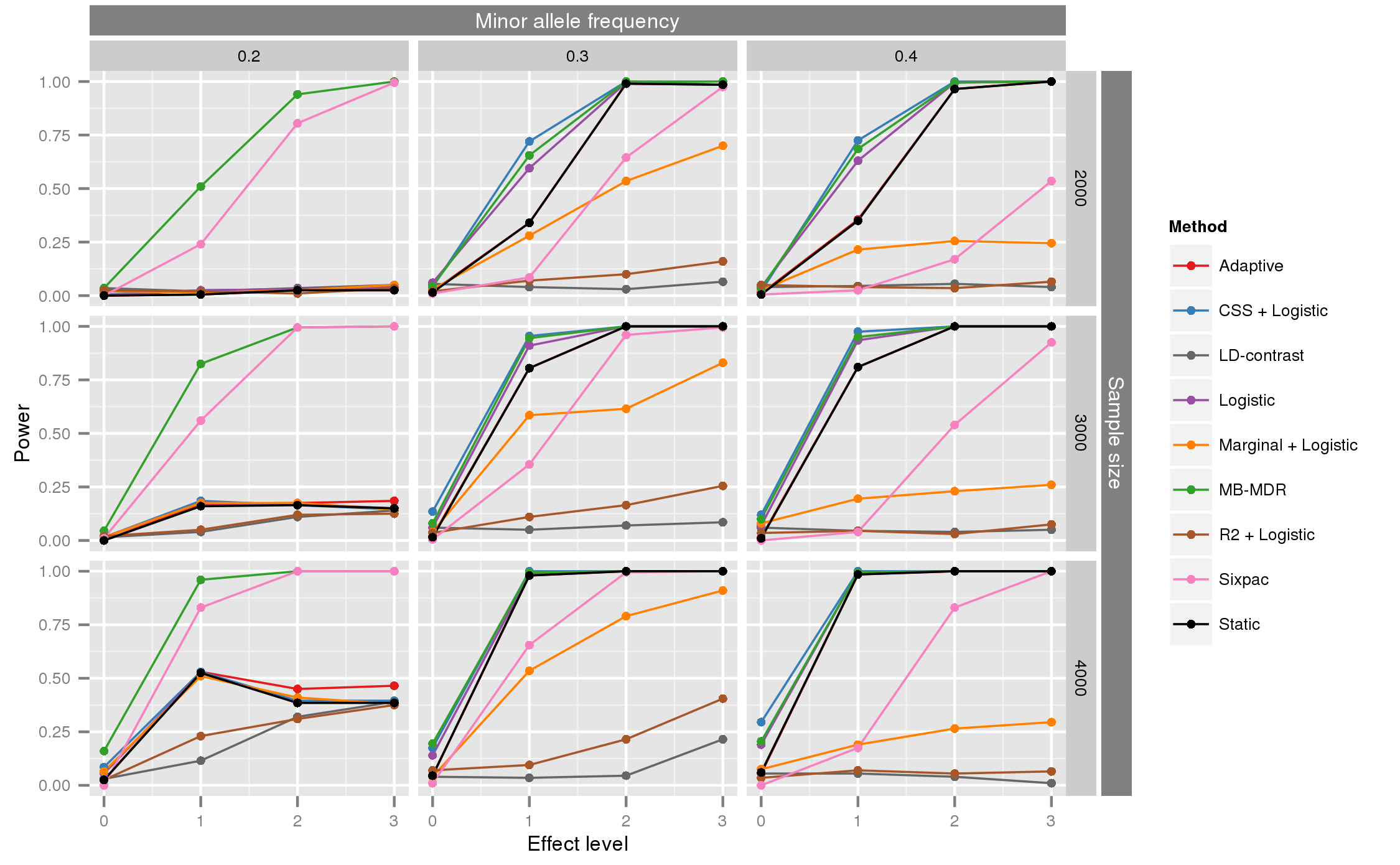

Supplement: S3 Fig — The x-axis is the effect level (i.e., categorized effect sizes, cf. S2 Text), and the y-axis is the power. The columns correspond to different minor allele frequencies. The rows correspond to different sample sizes under a balanced design e.g. 2000 indicates 2000 cases and 2000 controls. (Notice that the red line for the adaptive method is often hidden behind the black line for the static method.) (TIF) [file pgen.1005502.s005.tif]

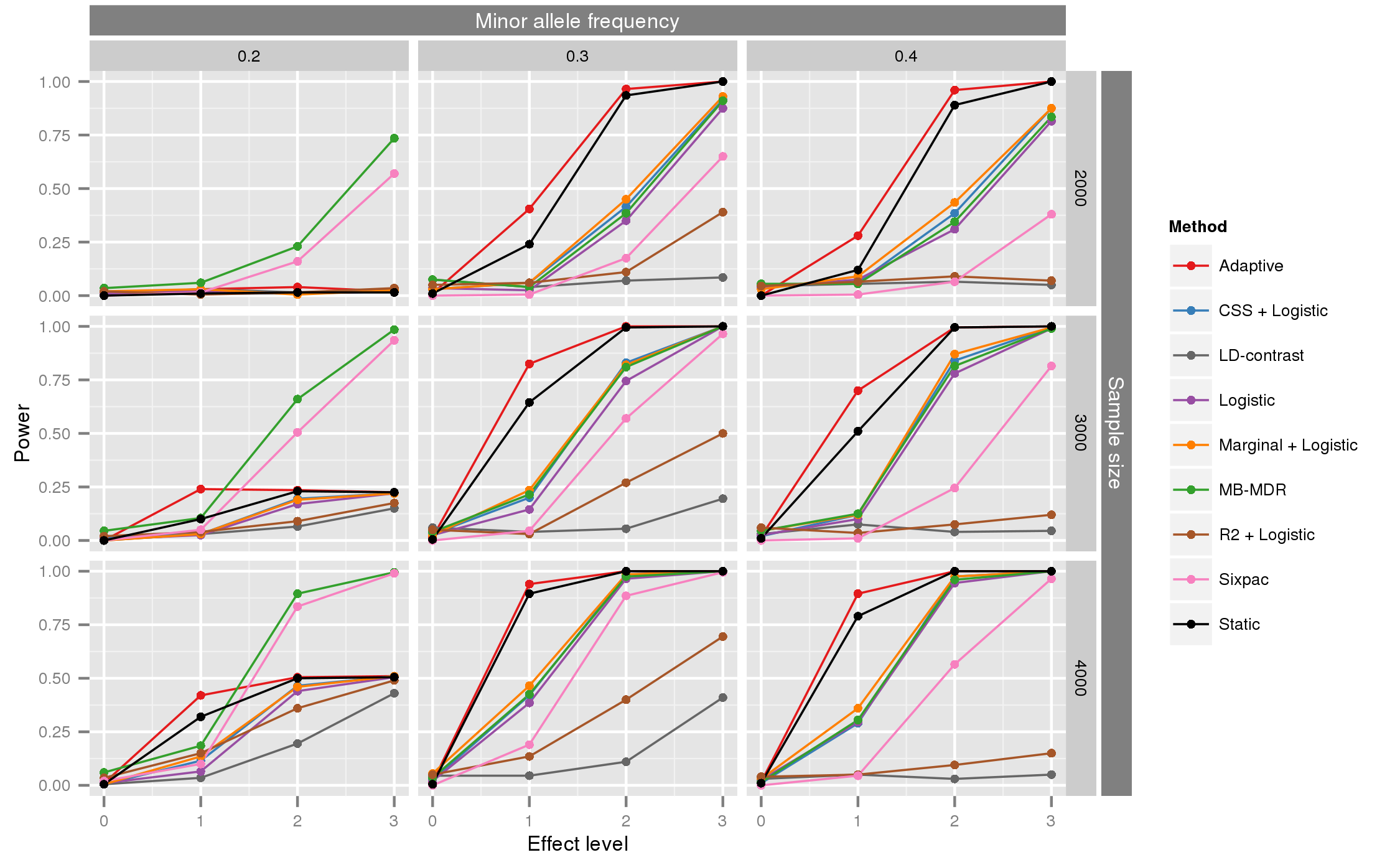

Supplement: S4 Fig — The x-axis is the effect level (i.e., categorized effect sizes, cf. S2 Text), and the y-axis is the power. The columns correspond to different minor allele frequencies. The rows correspond to different sample sizes under a balanced design e.g. 2000 indicates 2000 cases and 2000 controls. (TIF) [file pgen.1005502.s006.tif]

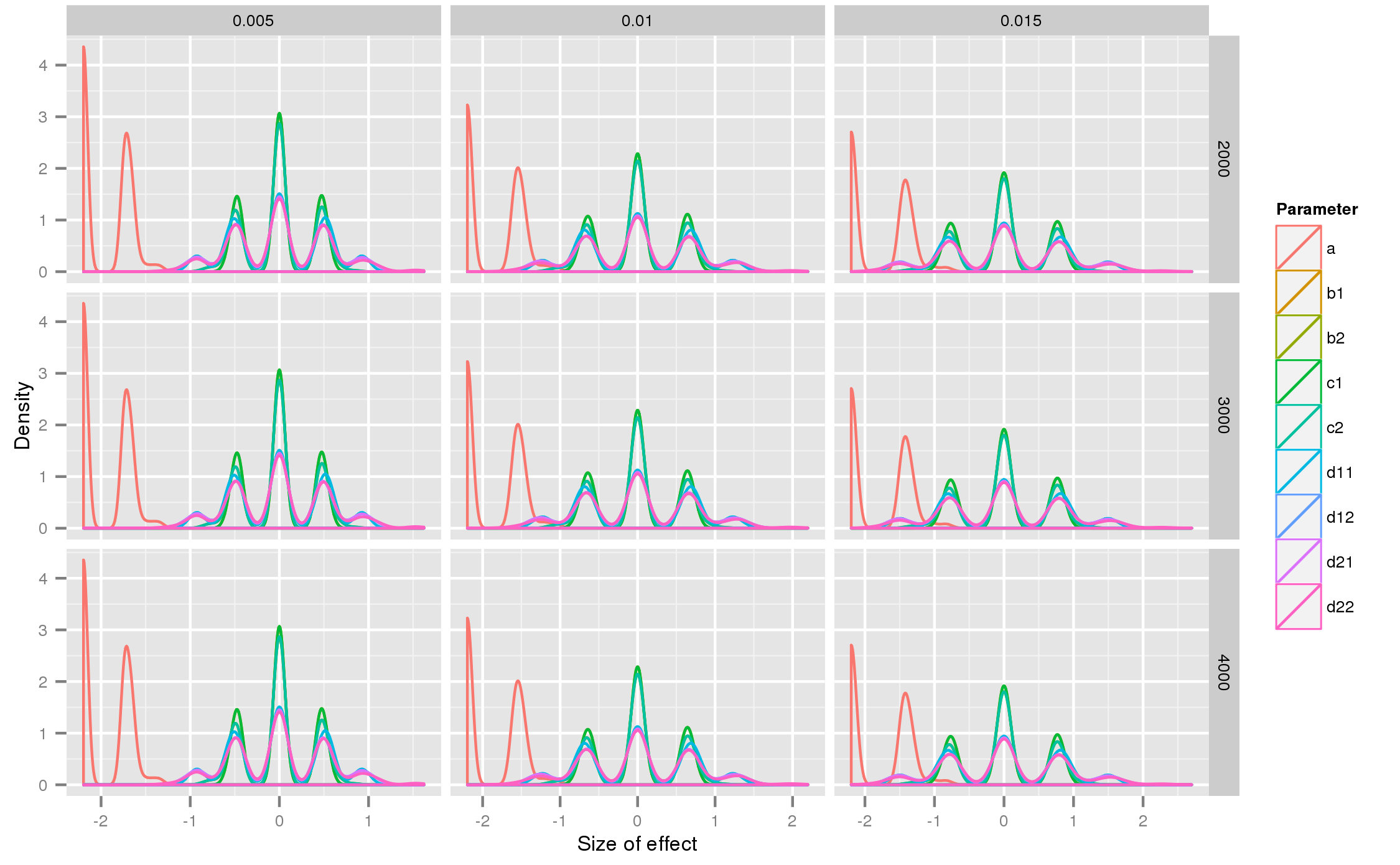

Supplement: S7 Fig — The rows correspond to sample sizes, and the columns to heritabilities. The differently colored lines correspond to the estimated density of different parameters in the models. The label “a” refers to the intercept α, “b1” and “b2” to the main effects of the first variant β 1 and β 2, “c1” and “c2” to the main effects of the second variant γ 1 and γ 2, and “d11”, “d12”, “d21” and “d22” to the interaction effects δ 11, δ 12, δ 21 and δ 22. (TIF) [file pgen.1005502.s009.tif]

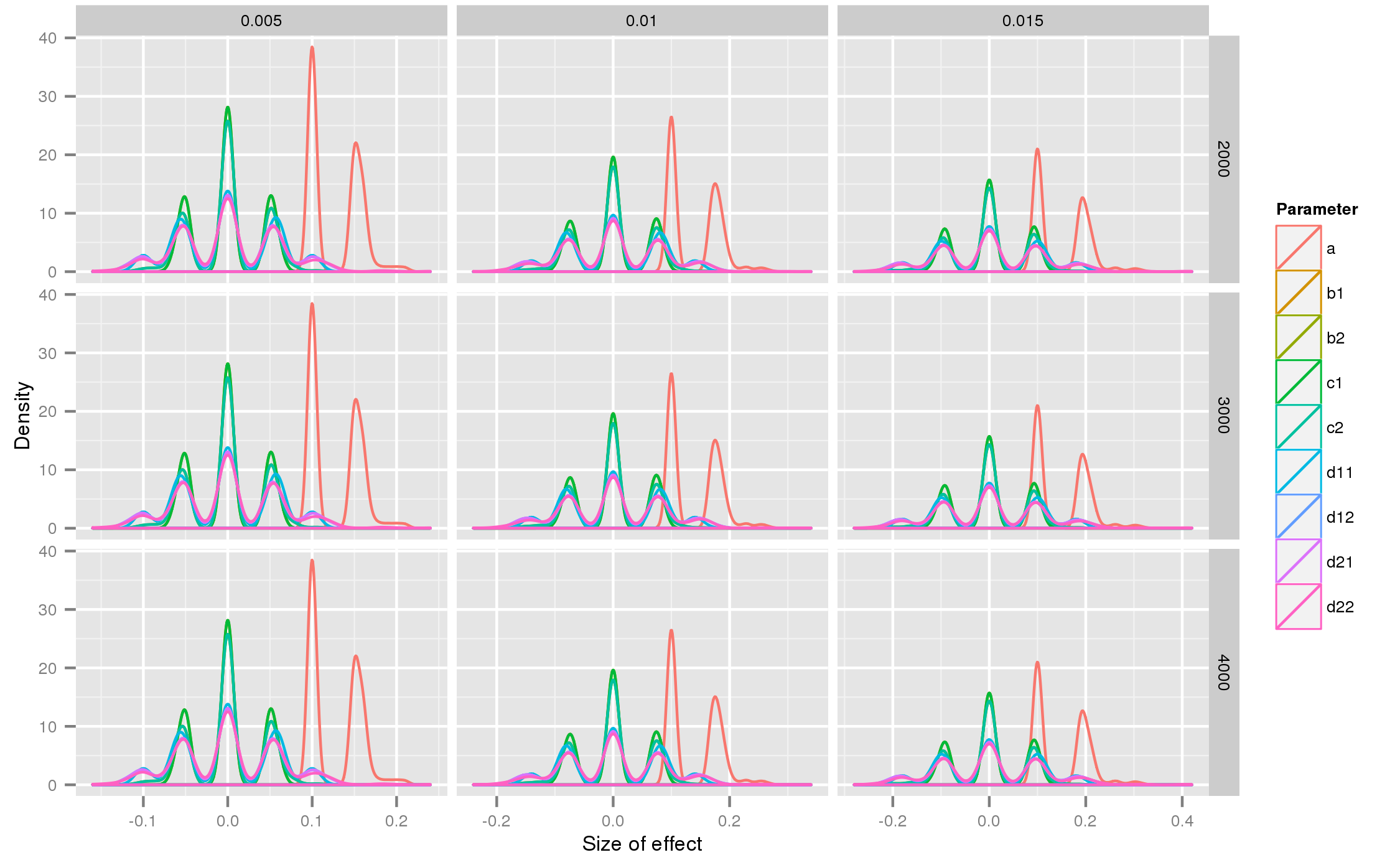

Supplement: S8 Fig — The rows correspond to sample sizes, and the columns to heritabilities. The differently colored lines correspond to the estimated density of different parameters in the models. The label “a” refers to the intercept α, “b1” and “b2” to the main effects of the first variant β 1 and β 2, “c1” and “c2” to the main effects of the second variant γ 1 and γ 2, and “d11”, “d12”, “d21” and “d22” to the interaction effects δ 11, δ 12, δ 21 and δ 22. (TIF) [file pgen.1005502.s010.tif]

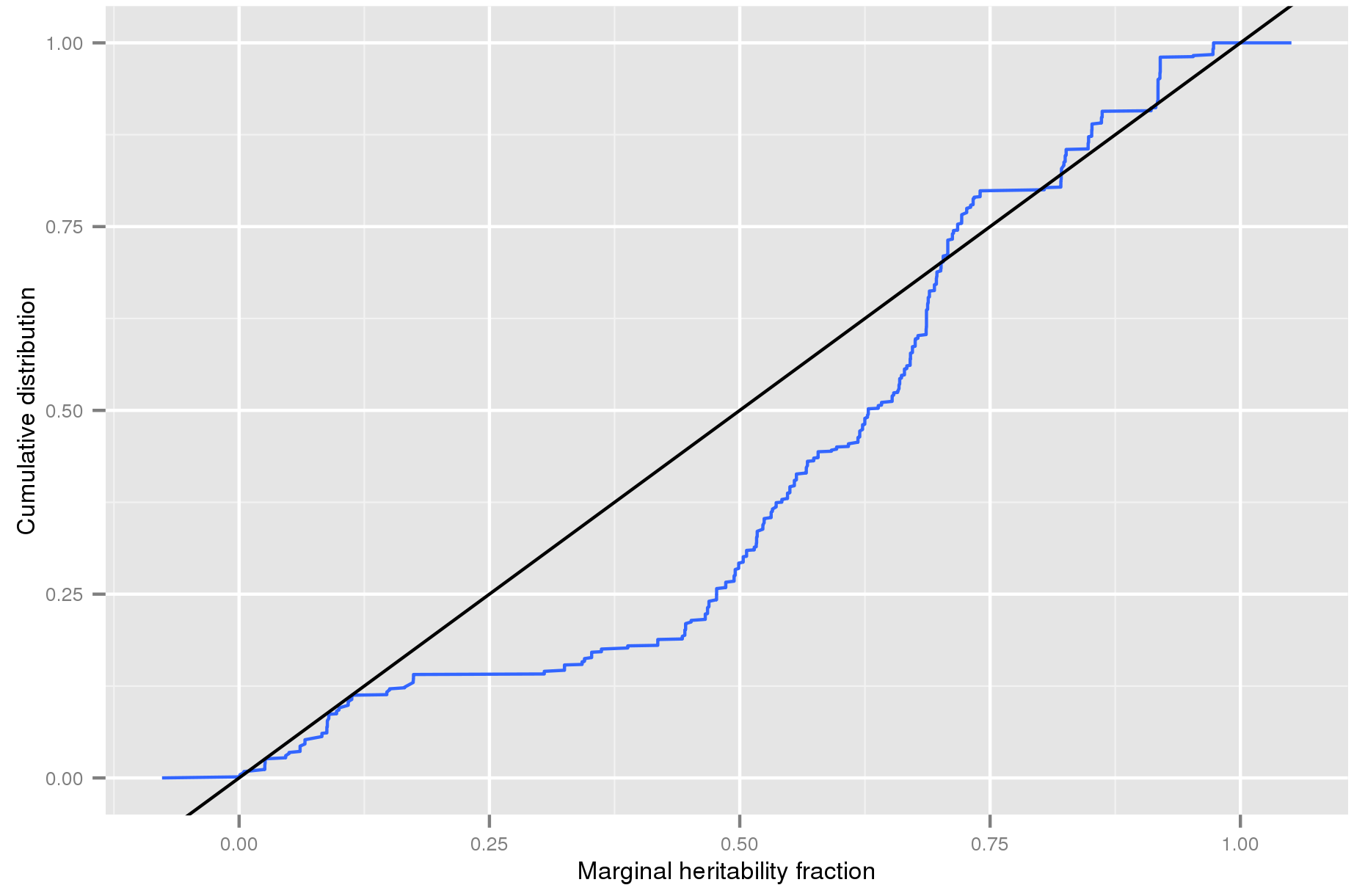

Supplement: S9 Fig — The x-axis represents a threshold for the marginal heritability fraction, and y-axis the fraction models with a marginal heritability fraction greater than this threshold. The blue line represents the empirical cumulative distribution function. The black line represents the cumulative distribution for a uniform distribution. The marginal heritability fraction was computed as the sum of the marginal heritabilities divided by the total heritability. (TIF) [file pgen.1005502.s011.tif]
