# Supplementary material for: Discovering Genetic Interactions in Large-Scale Association Studies by Stage-wise Likelihood Ratio Tests
Source: PLoS Genet. 2015 Sep 24;11(9):e1005502. doi: 10.1371/journal.pgen.1005502 (PMC4581725; doi:10.1371/journal.pgen.1005502)
Supplement: S1 Table — A list of the top variants in each of the published CAD loci associated in large-scale meta analyses [2], that are also present on the IBC-chip. (PDF) [file pgen.1005502.s012.pdf]

SNP	Chromosome	Position	Locus
rs11206510	1	55268627	PCSK9
rs646776	1	109620053	CELSR2
rs3008621	1	220870669	MIA3
rs17465637	1	220890152	MIA3
rs4299376	2	43926080	ABCG8
rs2706399	5	131895601	IL5
rs3798220	6	160881127	LPA
rs10455872	6	160930108	LPA
rs11556924	7	129450732	ZC3HC1
rs17321515	8	126555591	TRIB1
rs4977574	9	22088574	CDKN2B-AS1
rs1333049	9	22115503	CDKN2B-AS1
rs501120	10	44073873	LINC00841,LOC100130539
rs2246942	10	90994866	LIPA
rs3824755	10	104585839	CYP17A1
rs3184504	12	110368991	SH2B3
rs4773144	13	109758713	COL4A2
rs46522	17	44343596	UBE2Z
