# Supplementary material for: Discovering Genetic Interactions in Large-Scale Association Studies by Stage-wise Likelihood Ratio Tests
Source: PLoS Genet. 2015 Sep 24;11(9):e1005502. doi: 10.1371/journal.pgen.1005502 (PMC4581725; doi:10.1371/journal.pgen.1005502)
Supplement: S3 Table — Variant pairs that displayed significant interaction on at least one scale for each strategy in the original analysis of the PROCARDIS cohort are listed. The intercept is α. The main effects are β 1, β 2, γ 1, and γ 2. The interaction parameters are δ 11, δ 12, δ 21, and δ 22. LR is the likelihood ratio which measures the degree of evidence for the interaction model compared to the additive model on the logistic scale. (PDF) [file pgen.1005502.s014.pdf]

**All vs all strategy**			Main effects					Interaction			
SNP 1	SNP 2	α	β_1	β_2	γ_1	γ_2	δ_{11}	δ_{12}	δ_{21}	δ_{22}	LR
rs3917245	rs1412832	-2.034	-0.3034	-0.7309	-0.4216	-0.469	0.4372	0.1782	0.6209	0.02367	15.19
rs4846770	rs518394	-2.225	0.01927	0.1741	0.09202	-0.2434	-0.2973	-0.2748	-0.7592	-0.08044	15.24
rs17163313	rs518394	-2.229	0.01386	0.1651	0.08441	-0.2509	-0.2936	-0.2738	-0.7272	-0.06035	14.41
rs17163313	rs2069418	-2.226	0.05987	0.1264	0.1091	-0.2537	-0.3779	-0.2891	-0.6341	-0.07263	13.93
rs2378584	rs518394	-2.228	0.01937	0.1589	0.08839	-0.2487	-0.297	-0.276	-0.7229	-0.06119	14.4
rs4846770	rs2069418	-2.223	0.0644	0.1379	0.1189	-0.2451	-0.3793	-0.2897	-0.6696	-0.09414	14.62
rs17163301	rs518394	-2.228	0.0194	0.1587	0.0886	-0.2483	-0.3	-0.2667	-0.7227	-0.06135	14.32
**CAD loci vs all strategy**			Main effects					Interaction			
SNP 1	SNP 2	α	β_1	β_2	γ_1	γ_2	δ_{11}	δ_{12}	δ_{21}	δ_{22}	LR
rs17465637	rs518394	-2.258	0.06602	0.1875	0.08817	-0.2203	-0.3045	-0.289	-0.769	-0.3028	13.42
**HumanNet strategy**			Main effects					Interaction			
SNP 1	SNP 2	α	β_1	β_2	γ_1	γ_2	δ_{11}	δ_{12}	δ_{21}	δ_{22}	LR
rs4694178	rs583104	-2.327	0.1504	0.03143	-0.2108	0.3941	-0.1118	-1.37	0.1728	-1.305	21.86
rs4694178	rs602633	-2.33	0.1646	0.05609	-0.1859	0.5106	-0.1643	-1.495	0.1006	-1.367	22.46
