# Supplementary material for: Discovering Genetic Interactions in Large-Scale Association Studies by Stage-wise Likelihood Ratio Tests
Source: PLoS Genet. 2015 Sep 24;11(9):e1005502. doi: 10.1371/journal.pgen.1005502 (PMC4581725; doi:10.1371/journal.pgen.1005502)
Supplement: S4 Table — The MAF1 and MAF2 columns denote the minor allele frequency for variant 1 and variant 2 respectively. The counts n ij is the number of individuals with genotype i at the first variant and genotype j at the second variant. (PDF) [file pgen.1005502.s015.pdf]

Discovery												
**SNP 1**	**SNP 2**	**MAF 1**	**MAF 2**	n_{00}	n_{01}	n_{02}	n_{10}	n_{11}	n_{12}	n_{20}	n_{21}	n_{22}
rs4846770	rs518394	0.283	0.423	1127	1580	626	910	1219	485	184	240	106
rs17163313	rs518394	0.282	0.423	1133	1585	627	905	1213	481	181	239	105
rs3917245	rs1412832	0.226	0.293	1967	1571	354	1160	923	207	154	148	28
rs17163313	rs2069418	0.282	0.431	1115	1588	664	885	1222	502	174	239	113
rs2378584	rs518394	0.282	0.423	1134	1584	627	904	1217	483	183	239	106
rs4846770	rs2069418	0.283	0.431	1110	1582	663	890	1229	506	177	240	114
rs17163301	rs518394	0.282	0.423	1134	1584	627	904	1217	484	183	239	106
rs4694178	rs583104	0.449	0.204	1261	629	82	2047	1056	131	812	432	59
rs4694178	rs602633	0.449	0.192	1297	599	76	2116	988	121	839	410	52
rs17465637	rs518394	0.271	0.423	1174	1631	644	877	1184	479	170	226	94
**Replication**												
**SNP 1**	**SNP 2**	**MAF 1**	**MAF 2**	n_{00}	n_{01}	n_{02}	n_{10}	n_{11}	n_{12}	n_{20}	n_{21}	n_{22}
rs4694178	rs602633	0.485	0.153	1115	246	98	760	309	47	1066	182	91
