# Supplementary material for: Discovering Genetic Interactions in Large-Scale Association Studies by Stage-wise Likelihood Ratio Tests
Source: PLoS Genet. 2015 Sep 24;11(9):e1005502. doi: 10.1371/journal.pgen.1005502 (PMC4581725; doi:10.1371/journal.pgen.1005502)
Supplement: S5 Table — The weight for a stage i ∈ [4] is indicated by w i. The list is sorted in ascending order by the power obtained for the static stage-wise method and the weight combination selected for all remaining analyses in this paper is indicated by bold face. (PDF) [file pgen.1005502.s016.pdf]

Weights				Power	
w_1	w_2	w_3	w_4	Power adaptive	Power static
0.2	0.1	0.5	0.2	0.691	0.597
0.3	0.2	0.1	0.4	0.696	0.621
0.1	0.4	0.3	0.2	0.729	0.631
0.1	0.4	0.4	0.1	0.703	0.633
0.2	0.5	0.1	0.2	0.730	0.633
0.1	0.6	0.1	0.2	0.711	0.635
0.1	0.4	0.1	0.4	0.727	0.639
0.5	0.1	0.3	0.1	0.716	0.639
0.1	0.7	0.1	0.1	0.709	0.640
0.4	0.1	0.3	0.2	0.717	0.640
0.2	0.2	0.5	0.1	0.730	0.640
0.1	0.1	0.3	0.5	0.724	0.640
0.2	0.2	0.4	0.2	0.733	0.643
0.4	0.3	0.1	0.2	0.728	0.643
0.2	0.3	0.3	0.2	0.727	0.645
0.3	0.2	0.4	0.1	0.716	0.646
0.2	0.1	0.3	0.4	0.722	0.646
0.1	0.2	0.6	0.1	0.691	0.646
0.2	0.6	0.1	0.1	0.708	0.648
0.1	0.3	0.4	0.2	0.721	0.649
0.5	0.1	0.1	0.3	0.723	0.649
0.3	0.1	0.5	0.1	0.736	0.650
0.4	0.2	0.1	0.3	0.727	0.650
0.2	0.3	0.4	0.1	0.729	0.651
0.1	0.3	0.1	0.5	0.751	0.652
0.1	0.2	0.3	0.4	0.737	0.654
0.1	0.1	0.2	0.6	0.752	0.654
0.1	0.4	0.2	0.3	0.734	0.654
0.3	0.5	0.1	0.1	0.738	0.654
0.25	0.25	0.25	0.25	0.722	0.656
0.3	0.4	0.1	0.2	0.730	0.660
0.3	0.4	0.2	0.1	0.732	0.660
0.6	0.1	0.2	0.1	0.726	0.660
0.4	0.1	0.4	0.1	0.727	0.661
0.4	0.4	0.1	0.1	0.738	0.662
0.1	0.2	0.4	0.3	0.730	0.662
0.3	0.3	0.3	0.1	0.713	0.663
0.1	0.1	0.6	0.2	0.750	0.663
0.1	0.5	0.1	0.3	0.766	0.664
0.1	0.3	0.5	0.1	0.737	0.664
0.1	0.1	0.7	0.1	0.736	0.665
0.2	0.1	0.6	0.1	0.722	0.666
0.3	0.3	0.2	0.2	0.743	0.666
0.1	0.3	0.2	0.4	0.753	0.667
0.1	0.5	0.3	0.1	0.742	0.668
0.5	0.2	0.1	0.2	0.732	0.668
0.2	0.4	0.3	0.1	0.721	0.669

0.4	0.2	0.2	0.2	0.758	0.670
0.2	0.5	0.2	0.1	0.756	0.670
0.1	0.6	0.2	0.1	0.744	0.671
0.7	0.1	0.1	0.1	0.730	0.671
0.1	0.1	0.1	0.7	0.735	0.672
0.2	0.2	0.1	0.5	0.761	0.672
0.3	0.2	0.3	0.2	0.716	0.672
0.3	0.1	0.2	0.4	0.717	0.672
0.1	0.2	0.5	0.2	0.767	0.673
0.5	0.3	0.1	0.1	0.728	0.673
0.1	0.1	0.5	0.3	0.737	0.675
0.3	0.1	0.3	0.3	0.727	0.675
0.2	0.4	0.2	0.2	0.740	0.676
0.2	0.2	0.2	0.4	0.748	0.677
0.2	0.3	0.1	0.4	0.733	0.677
0.3	0.1	0.1	0.5	0.739	0.679
0.2	0.1	0.2	0.5	0.759	0.680
0.1	0.5	0.2	0.2	0.745	0.682
0.5	0.2	0.2	0.1	0.742	0.683
0.1	0.2	0.2	0.5	0.741	0.683
0.1	0.1	0.4	0.4	0.756	0.685
0.6	0.2	0.1	0.1	0.741	0.685
0.3	0.1	0.4	0.2	0.779	0.685
0.2	0.1	0.1	0.6	0.768	0.686
0.4	0.3	0.2	0.1	0.747	0.688
0.2	0.2	0.3	0.3	0.745	0.688
0.1	0.2	0.1	0.6	0.747	0.689
0.4	0.1	0.1	0.4	0.758	0.690
0.6	0.1	0.1	0.2	0.711	0.691
0.4	0.2	0.3	0.1	0.761	0.691
0.3	0.2	0.2	0.3	0.763	0.693
0.3	0.3	0.1	0.3	0.793	0.693
0.4	0.1	0.2	0.3	0.743	0.695
0.2	0.1	0.4	0.3	0.773	0.701
0.5	0.1	0.2	0.2	0.769	0.706
0.2	0.4	0.1	0.3	0.770	0.708
0.2	0.3	0.2	0.3	0.756	0.711
**0.1**	**0.3**	**0.3**	**0.3**	**0.809**	**0.723**
