# Supplementary material for: Discovering Genetic Interactions in Large-Scale Association Studies by Stage-wise Likelihood Ratio Tests
Source: PLoS Genet. 2015 Sep 24;11(9):e1005502. doi: 10.1371/journal.pgen.1005502 (PMC4581725; doi:10.1371/journal.pgen.1005502)
Supplement: S6 Table — The value p ij denotes the penetrance for genotype ij. The rows correspond to the sequence of effect sizes used, sorted in ascending order of the effect size. (PDF) [file pgen.1005502.s017.pdf]

Effect level	p_{00}	p_{01}	p_{02}	p_{10}	p_{11}	p_{12}	p_{20}	p_{21}	p_{22}
0	0.1000	0.1000	0.1000	0.1000	0.1100	0.1100	0.1000	0.1100	0.1100
1	0.1000	0.1000	0.1000	0.1000	0.1200	0.1200	0.1000	0.1200	0.1200
2	0.1000	0.1000	0.1000	0.1000	0.1300	0.1300	0.1000	0.1300	0.1300
3	0.1000	0.1000	0.1000	0.1000	0.1400	0.1400	0.1000	0.1400	0.1400
