# Supplementary material for: Discovering Genetic Interactions in Large-Scale Association Studies by Stage-wise Likelihood Ratio Tests
Source: PLoS Genet. 2015 Sep 24;11(9):e1005502. doi: 10.1371/journal.pgen.1005502 (PMC4581725; doi:10.1371/journal.pgen.1005502)
Supplement: S7 Table — The value p ij denotes the penetrance for genotype ij. The rows correspond to the sequence of effect sizes used, sorted in ascending order of the effect size. (PDF) [file pgen.1005502.s018.pdf]

Effect level	p_{00}	p_{01}	p_{02}	p_{10}	p_{11}	p_{12}	p_{20}	p_{21}	p_{22}
0	0.1000	0.1000	0.1000	0.1000	0.1500	0.1500	0.1000	0.1500	0.1500
1	0.1000	0.1000	0.1000	0.1000	0.1700	0.1700	0.1000	0.1700	0.1700
2	0.1000	0.1000	0.1000	0.1000	0.1900	0.1900	0.1000	0.1900	0.1900
3	0.1000	0.1000	0.1000	0.1000	0.2100	0.2100	0.1000	0.2100	0.2100
