# Supplementary material for: Discovering Genetic Interactions in Large-Scale Association Studies by Stage-wise Likelihood Ratio Tests
Source: PLoS Genet. 2015 Sep 24;11(9):e1005502. doi: 10.1371/journal.pgen.1005502 (PMC4581725; doi:10.1371/journal.pgen.1005502)
Supplement: S8 Table — The GLMS corresponds to the null models used in the stage-wise methodology. The intercept is α. The main effects are β 1, β 2, γ 1, and γ 2. (PDF) [file pgen.1005502.s019.pdf]

**Model**	Link	α	β_1	β_2	γ_1	γ_2
No association	Logit	-2.19	0	0	0	0
Single	Logit	-2.19	0.46	0.46	0	0
Double:identity	Identity	0.1	0.2	0.2	0.2	0.2
Double:log	Log	-2.3	0.4	0.4	0.4	0.4
Double:odds	Odds	0.1	0.3	0.3	0.3	0.3
Double:logit	Logit	-2.19	0.5	0.5	0.5	0.5
Double:log-compl	Log-complement	-0.1	-0.1	-0.1	-0.1	-0.1
